# Success rates of American clinical oncology trials by geographic factors

**DOI:** 10.1038/s41598-026-39609-x

**Published:** 2026-02-11

**Authors:** Sumeet Patiyal, Alejandro A. Schäffer

**Affiliations:** Cancer Data Science Laboratory, Center for Cancer Research, National Cancer Institute, National Institutes of Health, Bethesda, MD 20892 USA

**Keywords:** Cancer clinical trials, Clinical trial outcomes, Geographic analysis, Median income, Cancer, Health care, Medical research, Oncology

## Abstract

We aim to evaluate relationships between the locations and success rates of United States oncology clinical trials by analyzing geographic features such as median income and proportion of practicing oncologists by ZIP code. A dataset of 15,658 trials with at least one site in the 50 states or the District of Columbia was collected by integrating trial outcomes curated in Trialtrove, locations from ClinicalTrials.gov, incomes from the U.S. Census, oncologist locations from the Center for Medicare Services and other sources. We found that ZIP codes in which trials are conducted are skewed away from lower median incomes (*p* = 4.05e-08, Kolmogorov-Smirnov test). When ZIP codes were partitioned into lower, middle and upper terciles of median income, success rates by ZIP code were significantly lower in the low-income range relative to the middle- and higher-income ranges (lowest vs. highest *p* < 0.0001, OR 1.13 [1.10–1.16]). Trials conducted in more ZIP codes are significantly more likely to succeed than trials conducted in fewer ZIP codes. We identified candidate ZIP codes with sufficient patient populations and practicing oncologists to support clinical oncology trials but have never had a trial previously.

**Supplementary Information:**

The online version contains supplementary material available at 10.1038/s41598-026-39609-x.

## Introduction

 Geography is relevant to the availability of clinical trials and recruitment of patients to trials. At the individual patient level, worse patient outcomes are associated with geographic characteristics, including rural locations and further distance to travel^1–6^, areas with economic deprivation^7,8^ and sites that treat fewer patients per unit of time^3,9^.

At the level of entire trials, however, the relevant past work has largely missed the opportunity to investigate in detail possible relationships between geography and outcome, especially within the U.S. Past studies investigating entire trials and geography focused on participation, patient eligibility and accessibility, rather than trial outcomes^2,4,6–8,10–19^. There have been many past studies investigating associations between trial characteristics and trial outcomes and some of these studies included geographic variables among a much larger set of variables. The relevant previous studies considered three types of outcomes: transition from one phase to the next^20–25^, termination vs. completion^26–31^, and other outcomes^32–37^. The outcome of “transition from one phase to the next” is an outcome about treatments not about individual trials. The termination vs. completion outcome is popular for analyzing trials, because termination/completion is annotated in the publicly available ClinicalTrials.gov, albeit with many incomplete entries. In contrast, the studies using “other outcomes” typically require extensive curation but have more complete input data. Even if the input data were complete, a weakness of termination vs. completion is that it treats trials that were completed and failed to meet their primary endpoints as successful trials. Therefore, we instead used curated outcome data from Trialtrove, and we considered a trial successful if and only if the trial was as annotated as meeting its endpoint(s) (details in Methods).

In this study, we consider the following two-part research question: Do (a) the number of sites of a clinical trial or (b) the actual geographic locations have any statistical relationship to the success rate? Success rate is the proportion of trials that have a positive outcome. We hypothesized that success rate by ZIP code may be associated with median income, 1 – poverty rate, or proportion of practicing oncologists in the population of a ZIP code or county. We also hypothesized that geographic features would be able to improve prediction of clinical trial success. Having integrated the data for retrospective analysis, we also asked: could the integrated dataset be used to identify prospectively heretofore underutilized ZIP codes that have sufficient population and practicing oncologists to support a clinical trial? Because we are cancer researchers working in the United States, we restricted our analysis to interventional oncology trials with at least one site in the U.S.

We integrated six data sources (Trialtrove, National Library of Medicine ClinicalTrials.gov, U.S. Census American Community Survey, Centers for Medicare and Medicaid Services list of providers, Area Deprivation Index (ADI), and U.S. Department of Agriculture Rural Urban Continuum Codes, see Methods). Four of these were integrated in a recent study at the county level of ongoing clinical trials that overlaps topically with our ZIP code-level study^14^. We report on associations between clinical trial success and geographic-demographic variables such as median incomes and proportion of oncologists working in a ZIP code.

Using success rate of trials in a ZIP code as the dependent variable and ZIP codes with at least six trials, we found that ZIP codes in the lowest 10–33% range of median income have significantly lower trial success rates. Using instead the success/failure of an individual trial, we found that the inclusion of geography-related variables such as the median income by ZIP code of the trial site improves prediction of clinical trial success across multiple model designs. Further, to account for potential confounding by variables such as phase, sponsorship, and treatment type, we additionally performed multivariable logistic regression analyses, which were intended to assess adjusted associations for success of individual trials. In addition, we identify ZIP codes that have had no trials but have the most oncologists, which may be fruitful locations for future trials.

**Fig. 1:**
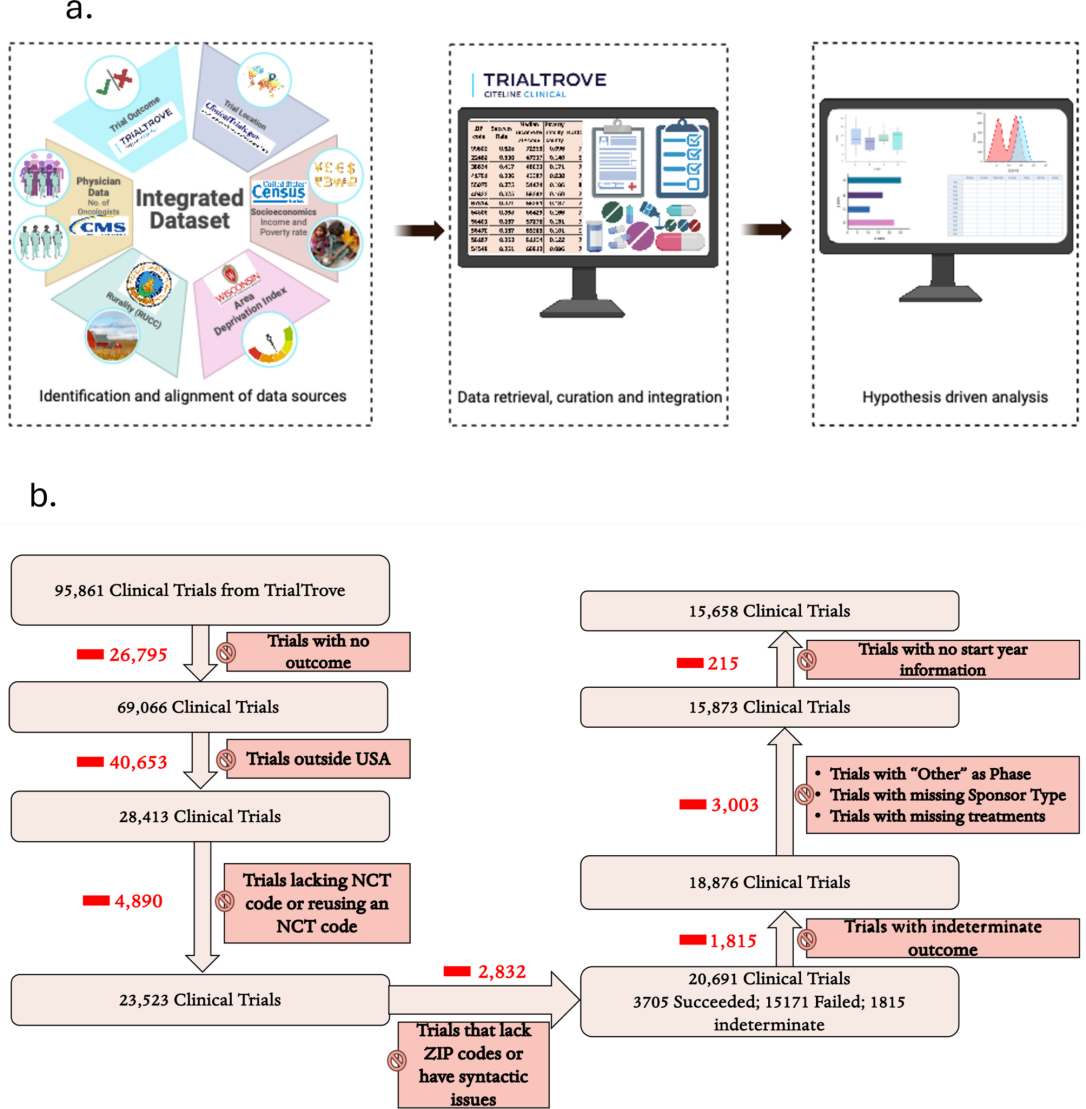
Study design and data curation. (**a**) Schematic diagram for the study design. We retrieved and curated the data from six sources, including trial outcomes (Trialtrove), trial locations (ClinicalTrials.gov), economic factors (U.S. Census), rurality (RUCC), area deprivation index (ADI), and physician data (Centers for Medicare and Medicaid Services). The integrated dataset of 15,658 U.S. oncology clinical trials served as the basis to investigate relationships between trial outcomes and geographic, economic, and healthcare-related factors. (**b**) Flowchart summarizing data curation from Trialtrove. Exclusions were applied for missing outcome data, non-U.S. trials, invalid or reused NCT codes, and incomplete ZIP code or metadata (e.g., missing start year, sponsor type, or treatment data). The final dataset comprises 15,658 trials, of which 3,367 succeeded, 12,291 failed.

## Results

### Overview of data analysis

Our analysis is in five parts and subsections 2.2 through 2.6. Subsection 2.2 describes the variance in median incomes, poverty rates, and oncologist proportion across sites. We show that using more locations for a trial is associated with a higher probability of success. In 2.3, we show the associations between ZIP codes (or counties) where trials are conducted and income, poverty rate, oncologist proportion, and rurality index. In subsection 2.4, we fit regression models to the success rate. In subsection 2.5, we present machine learning models to predict trial success based on non-geographic variables, and/or geographic variables. In subsection 2.6, we identify outliers in the data for (ZIP codes, oncologist proportion) and locations of past trials as possible locations for future trials.

### Included clinical trials and features

Depending on whether the trial start year was needed, either 15,873 trials or 15,658 trials had complete information and were used for analyses. Filtering steps are visualized in Fig. 1b and described in Methods and Supplementary Methods subsections SM2, SM3. Distributions of geographic and non-geographic variables are provided in Tables 1 and 2 and Supplementary Table S1. Distribution of trials by start year is shown in Figure S1.

**Table 1:** Descriptive statistics for geographic features. Statistics for geographic features for ZIP codes with six or more clinical trials, including median income by ZIP code, oncologist proportion, area deprivation indices (ADI) measures by ZIP code, poverty rate by county, and RUCC by county, to explore the characteristics of these high-trial-density regions. The ADI data are ranks, either within a state (STATERANK) or among States in the USA (NATRANK) and the RUCC data are based on a scoring system and hence the ADI and RUCC quantities are unitless. Outliers at the high end of oncologist proportion were filtered out for subsequent analyses (Methods).

Geographic features	Range	Median ± Standard Deviation
Number of distinct states	1–51	2 ± 8.71
Number of distinct ZIP codes	1–1155	2 ± 56.93
Median income by ZIP codes (dollars)	US$13,708–250,000	US$71,484 ± 30046.43
Median income Rank by ZIP codes (unitless)	25–30,576	17027.62 ± 8254.76
Number of oncologists in a ZIP code	0–507	64 ± 130.56
Oncologist proportion by ZIP code	0–0.79	0.002 ± 0.03
RUCC (by county) (unitless)	1–9	1 ± 0.35
Area Deprivation Index (ADI)		
State Rank (unitless)	1–10	2 ± 2.29
National Rank (unitless)	1–100	28 ± 20.99

**Table 2:** Descriptive statistics for non-geographic features. Distribution of successful and failed clinical trials across non-geographic features, categorized by trial phases (I, II, III), sponsor types (five categories), and treatment types (ten categories). Percentages of succeeded and failed trails are for single rows not for multiple rows.

Non-geographic feature	Succeeded	Failed	Total
Phase			
I	1325 (18.3%)	5911 (81.7%)	7236
II	1515 (21.9%)	5406 (78.1%)	6921
III	533 (31.1%)	1183 (68.9%)	1716
Sponsor			
Academic	1331 (17.8%)	6133 (82.2%)	7464
Industry	2733 (26.5%)	7580 (73.5%)	10,313
Government	767 (15.8%)	4095 (84.2%)	4862
Non-profit	33 (23.2%)	109 (76.7%)	142
Miscellaneous	38 (26.6%)	105 (73.4%)	143
Treatment type			
Immunotherapy	371 (31.3%)	813 (68.7%)	1184
Antibody	489 (26.2%)	1375 (73.8%)	1864
ADC	92 (28.5%)	231 (71.5%)	323
Radiotherapy	100 (18.5%)	441 (81.5%)	541
Hormone	126 (28.6%)	314 (71.4%)	440
Targeted	1175 (22.7%)	3991 (77.3%)	5166
Chemotherapy	488 (15.5%	2661 (84.5%)	3149
Immune-Other	293 (16.7%)	1464 (83.3%)	1757
Other	130 (13.3%)	850 (86.7%)	980
Supportive	109 (23.2%)	360 (76.8%)	469

Density plots of success rate and oncologist proportion for ZIP codes with six or more trials provide insight into their distributions (Supplementary Figure S2 and Supplementary Table S2). Success rate by ZIP codes highlights substantial variability, with a distribution skewed towards lower values (Supplementary Figure S2a).

The density plot for oncologist proportion by ZIP codes also reveals a skewed distribution with considerable variation (Supplementary Figure S2b). Most ZIP codes show very low oncologist proportion, including a peak at zero, indicating limited availability of oncologists. The peak at zero partly indicates that oncologists may travel from one ZIP code to another to conduct a trial. Distributions of other geographic variables by ZIP code are shown in Supplementary Figures S2c-h.

Non-geographic variables include trial phase, sponsor type, treatment type and start year. The distribution of trials across the phases revealed 7,236 (45.6%) trials were in Phase I and 6,921 (43.6%) in Phase II, compared with 1,716 (10.8%) in Phase III. Success rates by sponsor type are shown in Table 2. The most studied treatment type was Targeted therapy (32.54%), followed by Chemotherapy (19.84%) and Antibody (11.74%).

### Relationship between trial outcome and number of ZIP codes or states and median income

#### Analysis of trial locations, income, and poverty rate without outcomes

We first evaluated ZIP codes where trials have been conducted with respect to median income, 1- poverty rate and oncologist proportion irrespective of outcomes. The ZIP codes with at least one trial are significantly (Kolmogorov-Smirnov (K-S) test, *p* = 4.05e-08) skewed to higher median incomes (Supplementary Figure S3a). In contrast, the distributions of 1- poverty rate for all ZIP codes and ZIP codes with trials are not significantly different (Supplementary Figure S3b). The distributions of oncologist proportion for all ZIP codes and ZIP codes with trials (Supplementary Figure S3c) are not statistically significant either.

#### Correlation between success rate and economic and other factors

**Fig. 2:**
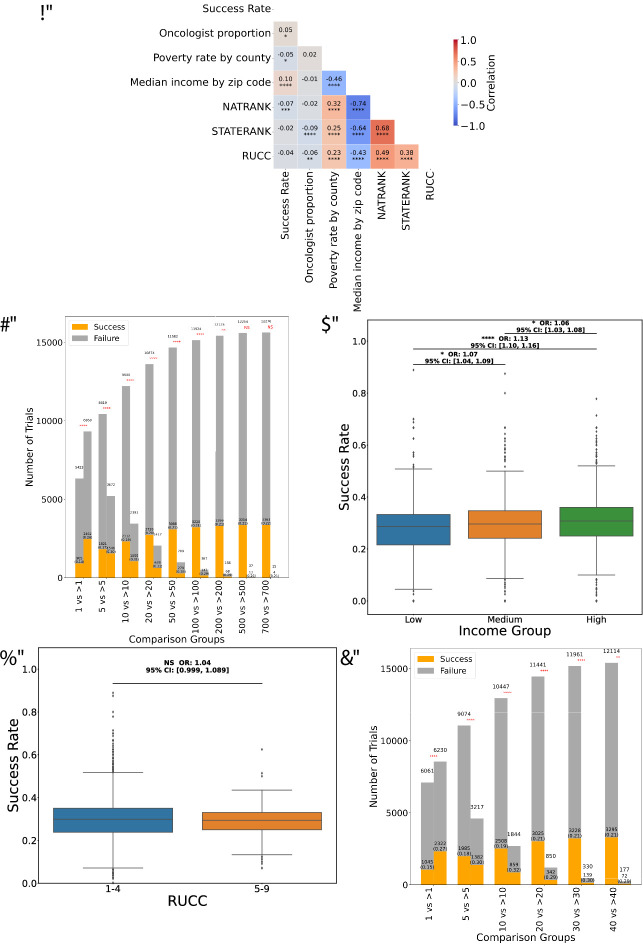
Overview of clinical trial success rates across various economic, geographic, and infrastructure dimensions. (**a**) Spearman correlation matrix illustrating the relationships between success rate and key variables, including oncologist proportion by ZIP code, 1-poverty rate by county, median income by ZIP code, ADI ranks (national and state) by ZIP code, and RUCC by county. (**b**) Comparison of success rates across trials involving fewer vs. more ZIP codes highlighting a trend of higher success rates with increased number of ZIP codes. In each test, represented by a pair of adjacent vertical bars, the two “comparison groups” are trials conducted in at most z ZIP codes vs. trials conducted in more than z ZIP codes, for different values of the threshold z along the x-axis. (**c**) Box plots of success rates for ZIP codes categorized into terciles of median income by ZIP code. Comparison between groups via Kruskal-Wallis test. (**d**) Box plots comparing trials conducted in RUCC 1–4 (urban/semi-urban) vs. RUCC 5–9 (rural) (Mann-Whitney test). (**e**) Success rates for trials spanning fewer versus more states. In each test, represented by a pair of adjacent vertical bars, the two “comparison groups” are trials conducted in at most s states vs. trials conducted in more than s states, for different values of the threshold s along the x-axis.

Success rate was positively correlated with both median income by ZIP code (ρ = 0.10, *p* < 0.0001) and oncologist proportion (ρ = 0.05, *p* < 0.05). Both the national and state ranks of the ADI were negatively correlated with success rate (NATRANK: ρ = −0.07, *p* < 0.001; STATERANK: ρ = −0.02, *p* > 0.05). Similarly, RUCC exhibited negative correlation with success rate (ρ = −0.04, *p* > 0.05) (Fig. 2a). These weak correlations suggest that there is some relationship between economic deprivation and trial success, but not a dominant relationship. We computed the same correlations separately for trials in phases I, II, III (Supplementary Figure S4). The correlations with success rate for phases I and III were very close to the correlations with success rate for all trials. However, for phase II trials, the correlation between success rate and oncologist proportion is negative, and the correlation between success rate and higher rurality (RUCC) is positive.

The poverty rate by county was positively correlated with the ADI NATRANK (ρ = 0.32, *p* < 0.0001), STATERANK (ρ = 0.25, *p* < 0.0001) and RUCC (ρ = 0.23, *p* < 0.0001). We hypothesized that areas with higher income levels, better healthcare infrastructure, and lower deprivation are more likely to see successful clinical trial results, while those facing higher poverty and deprivation face significant challenges in achieving successful outcomes. The clearest association is between success rate and median income (Fig. 2c, Supplementary Table S3), but the odds ratios for higher success rates in conjunction with higher median income in a ZIP code are only slightly above 1.0. Supplementary Table S3 summarizes all association analyses in which the dependent variable is the success rate of a ZIP code; the table shows the independent variable(s), the p-value if statistically significant, the odds ratio (OR), the 95% confidence interval for the odds ratio, and the number of the Figure in which the analysis is displayed.

#### Trends in trial outcome across ZIP codes

Threshold-based analysis comparing trials with *z* or fewer ZIP codes to trials with more than *z*, for *z* = 1, 5, 10, 20, 50, 100, 200, 500, and 700, showed that more ZIP codes for a trial is associated with a higher success probability (Fig. 2b). The results were similar for each trial phase, with the largest differences between fewer ZIP codes vs. more ZIP codes occurring in phase II (Supplementary Figure S5). We tried to fit polynomial curves to the data on number of ZIP codes and success rates but did not find a good fit (Supplementary Figure S6a, b).

Kruskal-Wallis tests revealed a significant overall difference in success rates across income groups (Fig. 2c and Supplementary Table S3, *p* = 1.18e-05). Post-hoc Dunn’s tests with Benjamini-Hochberg correction showed that both the low- vs. medium-income and low- vs. high-income comparisons were significant (FDR-adjusted *p* < 0.0001, *** annotation and *p* < 0.05, * annotation). We further analyzed the data using two alternative income partitions of 10:80:10 and 20:60:20 (Supplementary Figures S7a, b and Supplementary Table S3) obtaining similar results. The significant difference in success rates by median income is largely due to Phase II trials (Supplementary Figure S8, Supplementary Table S3). When stratified by 1-poverty rate categories, no statistically significant differences were observed (Supplementary Figure S9a, Supplementary Table S3). Success rates were higher in regions with at least one oncologist compared to regions with no oncologists (OR = 1.24, 95% CI: [1.19, 1.28]) (Supplementary Figure S9b, Supplementary Table S3).

Figure 2d and Supplementary Table S3 shows trial success rates in two RUCC categories: RUCC 1–4, representing urban to semi-urban areas, and RUCC 5–9, representing rural regions. The analysis reveals no statistically significant differences in success rates. We tested other RUCC categorizations (Supplementary Figure S10a, b, and Supplementary Table S3) and found that success rates remained stable at approximately 0.30.

We performed subgroup analysis of success rates across income groups stratified by trial start year, treatment type, and trial phase (Supplementary Figures S11, S12, S13 and Supplementary Table S3). There are no substantial changes among subgroups in the relationship between trial success and income groups.

#### Trends in trial outcome by number of participating states

Analysis of the number of states involved in trials yielded qualitatively similar results to the analysis of the number of ZIP codes. Trials with more states had significantly higher success rates than trials with fewer states in binary comparisons (Fig. 2e). This pattern did not change across trial phases (Supplementary Figure S14). However, we could not identify an optimal number of states (Supplementary Figure S6b.)

### Regression analysis using income, poverty and other features

To investigate the factors influencing the success rate, we analyzed seven variables: number of oncologists, oncologist proportion, median income by ZIP code, poverty rate by county, NATRANK, STATERANK, and RUCC. The analysis was conducted using ZIP codes with six or more trials. Initially, we performed linear regression with one variable at a time, followed by two-variable combinations (Supplementary Table S4). A Bonferroni correction diving the threshold by 5 was applied to account for dependencies between related variables, making an unadjusted p-value of < 0.01 statistically significant. Only median income by ZIP code and its combinations with other variables achieved significance after correction. We also applied linear and non-linear regression methods with median income by ZIP code paired with one additional variable and results are shown in Supplementary Table S5.

### Predicting clinical trial outcomes using geographic and non-geographic features

**Fig. 3:**
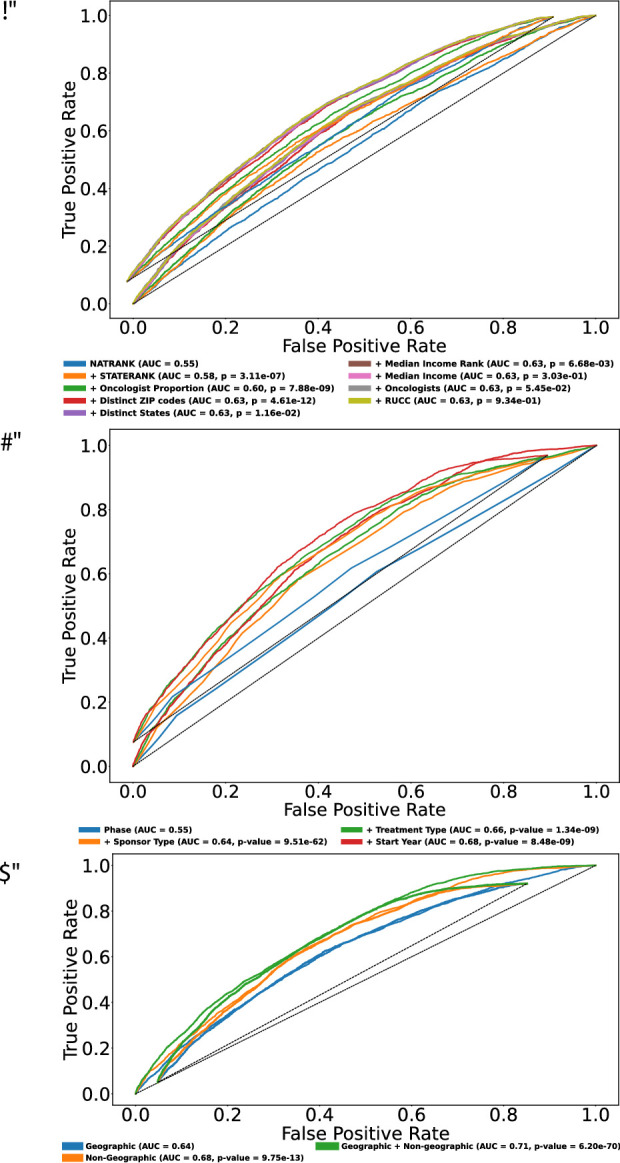
Receiver operator characteristic (ROC) curves demonstrating the significant impact of geographic features on model performance for predicting clinical trial outcomes. (**a**) Stepwise improvement in AUC with the inclusion of geographic features, starting with NATRANK (AUC = 0.55) and progressively increasing to 0.63 with the addition of STATERANK, oncologist proportion, distinct ZIP codes, distinct states, median income, and RUCC. DeLong’s test confirmed that these additions, particularly STATERANK (*p* = 3.11e − 07), Oncologist proportion (*p* = 7.88e − 09), Distinct ZIP Codes (*p* = 4.61e − 12), Distinct States (*p* = 1.16e-02), and Median Income Rank (*p* = 6.68e-03) are significant. (**b**) Sequential improvement with non-geographic features, beginning with Phase (AUC = 0.55), subsequent inclusion of Sponsor Type (AUC = 0.64, *p* = 9.51e − 62), Treatment Type (AUC = 0.66, *p* = 1.34e − 09), and Start Year (AUC = 0.68, *p* = 8.48e − 09) further improved predictive accuracy. (**c**) The combination of geographic and non-geographic features achieved the highest AUC of 0.71 compared to using non-geographic features alone (AUC = 0.68, *p* = 9.75e-13).

To predict clinical trial outcomes, we applied random forest classifiers using a geographic and/or non-geographic feature (Methods). The analysis of geographic features alone revealed a stepwise improvement in model performance as features were added in descending order of importance: ADI-based metrics, oncologist proportion, numbers of ZIP codes and states, median income rank and value. For only geographic features as measured by the area under the receiver operator characteristic curve, AUC = 0.63 (Fig. 3a). The p-values were calculated using DeLong’s test (Methods)^38^.

Non-geographic features were incorporated in the order: phase, sponsor type, start year (early vs. late) and yielded AUC = 0.68 (Fig. 3b). Combining geographic and non-geographic features yielded the highest AUC of 0.71. DeLong’s test confirmed that the combined feature set performed significantly better than using geographic or non-geographic features alone (*p* = 6.20e-70 and 9.75e-13, respectively) (Fig. 3c).

In addition to the prediction of trial success using random forests, we examined geographic and non-geographic features for confounding using multivariable logistic regression to assess their associations with trial outcome after adjustment for different features. In multivariable logistic regression analysis including geographic variables only, variable “[number of] Distinct states” was strongly associated with higher odds of success (OR = 1.04, *p* = 4.57e-36), while trials conducted in more rural settings had lower odds of success with slight significance (OR = 0.86, *p* = 0.025) as shown in Supplementary Table S6. Other geographic variables, including median income, oncologist density, and area deprivation indices, were not independently associated with success when considered jointly. In logistic regression analysis including only non-geographic features, industry sponsorship, higher trial phase, and trials started after 2010 were strongly associated with success. Combining both geographic and non-geographic features, “Distinct states” remained independently associated with higher odds of success (OR = 1.03, *p* = 3.26e-18), whereas trials conducted in more rural settings showed lower odds of success but was not significant (Supplementary Table S6). In contrast, median income, oncologist density, and area deprivation measures were not independently associated with success after adjusting for other trial features.

### Underutilized oncologist-dense sites for clinical trials

We searched for ZIP codes that had no clinical trials but did have oncologists to suggest sites for future trials. We required that at least one oncologist be present. In Table 3, we list locations with the highest proportion of oncologists but no past oncology clinical trials. One caveat is that there may be clinical trials in nearby ZIP codes.

**Table 3:** Candidate ZIP codes for future oncology trials. Top 20 sites with the highest proportion of oncologists but no oncology clinical trials. In this Table, ZIP codes were analyzed without applying a rurality or distance constraint, highlighting areas with substantial healthcare resources but no oncology trial activity. A ZIP code can be in multiple cities or counties. A City or County can have multiple ZIP codes.

ZIP code	#Trial	Oncologists	Population	Oncologist Proportion	City Name	State Name	County Name	NATRANK	STATERANK
75,766	0	144	26,128	0.005511	Jacksonville	Texas	Cherokee	96	10
21,156	0	60	362	0.165746	Upper Falls	Maryland	Baltimore	20	3
97,355	0	43	32,777	0.001312	Lebanon	Oregon	Linn	56	9
16,127	0	41	15,350	0.002671	Grove City	Pennsylvania	Mercer	81	8
30,152	0	40	43,865	0.000912	Kennesaw	Georgia	Cobb	17	1
94,702	0	37	17,473	0.002118	Berkeley	California	Alameda	5	3
85,037	0	34	51,987	0.000654	Phoenix	Arizona	Maricopa	49	7
20,707	0	33	36,352	0.000908	Laurel	Maryland	Prince George’s	34	6
28,037	0	33	24,834	0.001329	Denver	North Carolina	Lincoln	50	4
76,034	0	25	25,793	0.000969	Colleyville	Texas	Tarrant	8	1
84,631	0	22	2986	0.007368	Fillmore	Utah	Millard	43	8
11,102	0	22	27,069	0.000813	Astoria	New York	Queens	12	3
54,011	0	21	6925	0.003032	Ellsworth	Wisconsin	Pierce	64	6
70,301	0	20	47,429	0.000422	Thibodaux	Louisiana	Lafourche	83	7
19,602	0	20	17,953	0.001114	Reading	Pennsylvania	Berks	100	10
38,004	0	20	11,604	0.001724	Atoka	Tennessee	Tipton	49	3
64,063	0	19	21,232	0.000895	Lees Summit	Missouri	Jackson	57	4
30,019	0	19	46,553	0.000408	Dacula	Georgia	Gwinnett	28	2
89,011	0	18	33,600	0.000536	Henderson	Nevada	Clark	55	8
77,375	0	18	67,965	0.000265	Tomball	Texas	Harris	31	2

**Table 4:** Better candidates for future oncology clinical trials. The top 18 ZIP codes meeting the criteria of Tables 3 and the following three additional criteria. We added the requirements of a rurality threshold of RUCC ≥ 6, population at least 5000 but no NCI cancer center within 50 miles. ZIP codes are ranked in decreasing order by population. There May be other ZIP codes in the same City or County that did have a clinical trial.

ZIP code	Oncologists	Population	Oncologist Proportion	City Name	State Name	County Name	NATRANK	STATE RANK	RUCC
72,653	6	28,615	0.00021	Mountain Home	Arkansas	Baxter	78	5	7
75,455	9	26,816	0.000336	Mount Pleasant	Texas	Titus	77	8	7
75,766	144	26,128	0.005511	Jacksonville	Texas	Cherokee	96	10	6
28,779	5	18,421	0.000271	Sylva	North Carolina	Jackson	53	4	7
83,001	6	17,721	0.000339	Jackson	Wyoming	Teton	3	1	7
99,669	9	17,382	0.000518	Soldotna	Alaska	Kenai Peninsula	45	6	7
75,801	9	16,549	0.000544	Palestine	Texas	Anderson	70	7	7
36,426	5	15,932	0.000314	Brewton	Alabama	Escambia	95	9	6
31,023	5	14,404	0.000347	Eastman	Georgia	Dodge	70	10	7
65,708	9	13,114	0.000686	Monett	Missouri	Barry	85	8	6
56,031	5	11,861	0.000422	Fairmont	Minnesota	Martin	87	10	6
58,301	5	10,190	0.000491	Devils Lake	North Dakota	Ramsey	82	9	7
46,701	5	8091	0.000618	Albion	Indiana	Noble	69	5	6
64,759	5	7803	0.000641	Lamar	Missouri	Barton	92	9	8
72,031	14	7097	0.001973	Clinton	Arkansas	Van Buren	87	7	8
56,573	6	6733	0.000891	Perham	Minnesota	Otter Tail	63	8	6
83,544	5	6329	0.00079	Orofino	Idaho	Clearwater	69	9	8
74,441	6	5692	0.001054	Hulbert	Oklahoma	Cherokee	83	6	6

To incorporate other geographic constraints, we also prepared Table 4 that adds the following restrictions: RUCC, a measure of rurality, ≥ 6, ZIP code population at least 5000, and no NCI-designated Cancer Center within 50 miles. In Supplementary Table S7, we present a longer list of ZIP Codes, allowing for as few as 1 oncologist and any population. The requirements for RUCC ≥ 6 and population ≥ 5000 in Table 4 are difficult to satisfy simultaneously. Kirkwood and colleagues did a related analysis of ongoing clinical trials to find US counties that have suitable oncology care infrastructure and a paucity of trials that were recruiting at the time of their analysis^14^.

## Discussion

In this study, we assessed whether the geographic factors of sites for American oncology trials are associated with trial success. Trials are preferentially conducted and have higher success rates in middle to high income ZIP codes compared to lower income ZIP codes. (Supplementary Figure S3a, Fig. 2c, Supplementary Figure S7, Supplementary Table S3). Adding geographic features to a predictive model of trial outcome did significantly improve prediction. Additionally, we identified heretofore underserved locations for future oncology trials (Table 2a, b).

This study highlighted nuances of geographic features related to clinical trial success. Trials recruiting in more ZIP codes or more states have a higher success probability. It is interesting to compare our analysis of number of sites with one medium-scale (number of trials) study of large-scale (number of patients) trials conducted by the Southwestern Oncology Group (SWOG); they showed for such trials, outcomes are similar for patients in urban and rural areas^5^.

Most of the relevant prior studies were performed at the patient level rather than at the trial level, making it difficult to compare our results to prior work. Our findings statistically significant associations between geographic variables and clinical trial success in the U.S, but with small effect sizes. Our odds ratios for trial success when comparing higher median income ZIP codes to lower income ZIP codes are modest, in the range of 1.05 to 1.2 depending on how income levels are partitioned (Fig. 2c, Supplementary Figure S7, Supplementary Table S3). Our analysis draws on a sample nearly 10 times that of Hegge et al^22^., which may contribute to why their international study was negative about the role of geography. Based on their summary, it is likely that they included trials from any country and their geographic variable was defined at country level. If so, their coarser spatial granularity would not conflict with our results based on finer-grained ZIP code-level U.S. data.

Our finding that conducting trials in more ZIP codes and states is associated with higher probabilities of success can be compared to studies about the number of countries in international trials. Trials have been increasing international recruitment^12,16^, which makes it possible to test for an association between number of countries and trial success. Having more countries for a trial can help with recruitment^37^ and helps test whether the treatment is comparably effective in different ethnic populations. However, having more countries has been associated with problems in participant screening, in trial procedures, and trial duration^37^. Five studies gave mixed results about the merits of having more trial locations and the merits of some countries over other countries^20,23,24,33,37^ Our findings suggest that improving the breadth of patients recruited, in whatever geographic context, is associated with improved trial outcomes.

Rurality was not associated with trial-level success in any analysis, in contrast with some patient-level findings^1,3–6^ that found rurality to be a negative factor. Our findings underscore the potential for clinical trials to succeed whether they are conducted in urban or rural settings. One way to address prospectively the challenges described at the individual patient level in rural settings^2,11,19^ is to choose more trial sites in rural areas as we suggest in Tables 2a, b.

Our findings are actionable both in designing future oncology clinical trials in the USA and for reporting trial results. Trial designers should try to increase the number of locations for trials (Fig. 2b) and, where feasible, should try to arrange transportation for possible enrollees over a larger geographic area^15^. When adding locations, trial designers should favor either established locations that have had many trials and higher success rates in the past or underutilized locations with zero or few past trials and evidence of sufficient infrastructure for a trial (Tables 3 and 4). Trial designed should disfavor locations with many past trials and lower success rates. In addition, when reporting clinical trial results, the oncologists conducting the trial should ideally report results for each patient anonymized and include the trial location at which each patient has been treated^39–41^. Patient-by-patient reporting would allow many additional meta-analyses that are currently not possible. If trial designers are reluctant to report results for each patient, they should at least report results separately for each trial location and compare the results between locations.

Our study has limitations inherent to our goal of integrating multiple relevant datasets. First, we relied on Trialtrove, which requires a paid license. Second, we relied on ClinicalTrials.gov for which the geographic and other data are known to be incomplete^42,43^ Third, we analyzed data at the granularities of ZIP codes but we had to use poverty rates at the county level. Finer-grained analysis using modern geographic information systems (GIS) tools might yield more insights but data such as ADI is not available at a finer level of detail^15^. In particular, GIS tools have been used to quantify what proportion of the population lives within a certain travel distance or estimated commuting time to at least one ongoing oncology trial^14,15,44^. Both oncologists and patients may frequently travel from one ZIP Code to another to provide care or receive care. Fourth, various pieces of data we analyzed are snapshots from dates in 2022–2024 and differ today. Fifth, we were unable to assess how individual sites contributed to overall trial success or failure. For this, it would be ideal to have a dataset with response rates of patients at each trial site for each of the 15,658 trials. However, such granular data are not available outside of specific cooperative group analyses, such as SWOG trials^5^.

In conclusion, the main contribution of our study is to evaluate at the trial level how the number of sites and the geographic locations have relationships to the success rates of oncology clinical trials in the U.S. We did the evaluation by integrating data from Trialtrove, ClinicalTrials.gov, the U.S. Census and other sources. An overarching goal is to use retrospective data to provide feedback for oncologists in designing and deploying future clinical trials.

## Methods

### Geographic identifiers

We considered completed oncology clinical trials that were at least partly conducted in the United States. Since 1963, each address in the U.S. has been assigned a five- or nine-digit postal code called a Zone Improvement Plan (ZIP) code. We used five-digit ZIP codes; we did not use nine-digit ZIP codes for any analyses. We focused on ZIP codes as our geographic unit of analysis because ZIP codes allowed us to integrate some data sources, such as ADI, that we otherwise would not have been able to use. More information on how ZIP codes were obtained and used is in Supplementary Information subsection SM1. Some other studies analyzed at the level of counties^4,14,18^. However, many cities and counties have multiple ZIP codes and income often varies widely between different ZIP codes in the same county. A ZIP Code can be in multiple cities or counties, in which case we matched the ZIP code to the county with the most population in that ZIP code.

### Included clinical trials and data sources

Trials were included only if they had a clearly defined outcome and usable ZIP code information. For downstream analyses, we used trials with complete information for the considered variables.

As illustrated in Fig. 1a, we integrated the following six sources (details in Supplementary Information subsection SM2).

1. From Trialtrove via https://clinicalintelligence.citeline.com/trials/results, we obtained the list of all included interventional oncology trials, countries or territories where trials were conducted, and outcomes. Trialtrove distinguishes U.S. Territories (e.g., Puerto Rico) from the 50 states and the District of Columbia; therefore, “United States” refers only to the 50 states and the District of Columbia. Trialtrove is available by paid license, which limits us to publishing summary data. We and others have previously published large-scale studies based on Trialtrove despite this limitation^24,25,45,46^. We started with 23,531 completed trials.
2. From ClinicalTrials.gov, we obtained ZIP code location. After quality control, 20,691 among the 23,531 trials had usable locations with a ZIP code.
3. From the United States Census (data.census.gov), we obtained the following American Community Survey (ACS) data for the survey ending in 2022 including: population by ZIP code, poverty rate by county, and median income by ZIP code.
4. From the Centers for Medicare and Medicaid Services (https://data.cms.gov/provider-data/dataset/mj5m-pzi6), we obtained the list of oncologists practicing in each ZIP code. Combining number of oncologists and population from the Census, we computed oncologist proportion = (number of oncologists practicing in a ZIP Code)/(ZIP code population). The oncologist data were downloaded on May 8, 2024. We included all types of oncologists including radiation, hematology, surgical, etc.” “An oncologist may be listed multiple times in the CMS data with different addresses, and we counted each such entry as 1 oncologist even though the time of the oncologist must be split between the locations. We applied the 1.5*IQR (Inter-Quartile Range) heuristic to detect outliers^47^ among the highest oncologist proportion values and thereby eliminated 36 ZIP codes with oncologist proportions > 0.005783, in areas with many medical offices and few residents.
5. The Area Deprivation Index (ADI) from University of Wisconsin (https://www.neighborhoodatlas.medicine.wisc.edu/)^48^. The data publicly available are relative ranks of ZIP codes in a state (1 to 10) or in the U.S. (1 to 100); these are denoted STATERANK and NATRANK. Low ranks correspond to less deprived areas, while high ranks correspond to more deprived areas. ADI data were downloaded on July 31, 2024 and are annotated as being for the year 2022, which is consistent with 2022 being the end year of the ACS.
6. The Rural Urban Continuum Codes (RUCC) from the U. S. Department of Agriculture (https://www.ers.usda.gov/data-products/rural-urban-continuum-codes/) ranks each county from 1 (most urban) to 9 (most rural).

To aid data integration, we used data from the U.S. Department of Housing and Urban Development (HUD) via https://www.huduser.gov/apps/public/uspscrosswalk/login and published guidance^49,50^.

In total, 15,658 clinical trials could have data integrated. Start years range from 1966 to 2024, but only 12 trials started before 1991 and these may be data entry errors in Trialtrove. The large majority of trials started during 2005–2017 when the number of trials started per year was in the range 957–1094 (Supplementary Figure S1). After 2017, the number of started trials per year drops off because many trials have not been completed. The selection of sources and analysis methods was inspired by previous research. The idea to include the number of oncologists and the data source came from Levit et al.^4^. An early paper by Sateren et al.^51^ used the number of members of the American Society of Clinical Oncology as a proxy for the number of oncologists in a state. The idea to include poverty rates came from Unger et al.^7^. The idea of using the RUCC came from Unger et al.^5^. More information on data sources is in Supplementary Methods subsection SM2. More information on filtering steps to get to the final dataset is in Supplementary Methods subsection SM3.

###  Clinical trial outcomes

A trial was eligible (Fig. 1b) if there was an outcome in Trialtrove in the column “Trial Outcome(s)” and the outcome did not include “Completed, Outcome indeterminate”. A trial was considered successful if the outcome included the text “Completed, Early positive outcome” or “Completed, Positive outcome”; trials with any outcome other than the three quoted outcomes were unsuccessful. Past studies used a variety of definitions of trail success^23,32^. We followed the examples of several previous studies used commercially curated trial outcomes because the information on trial outcomes in ClinicalTrials.gov is incomplete and heterogeneous^25,32,34^.

### Primary variables for data analysis

*Geographic Features*: Geographic features include (number of) ZIP codes, (number of) states, median income by ZIP code, oncologist proportion, ADI, poverty rate by county, and RUCC. We selected “oncologist proportion”, “median income by ZIP code” and “poverty rate by county” containing the ZIP code to represent how well a ZIP code is served. When analyzing poverty, we display 1-poverty rate, which is expected to increase with median income. ZIP codes were categorized into three income groups based on median income: low, medium, and high with three different partitions. Details are in Supplementary Methods subsection SM4.

*Non-geographic features:* These include trial phase, sponsor type, treatment type, start year.

Trial phase was assigned to be Phase I = 1, Phase II = 2, or Phase III = 3. Sponsor type was categorized into five binary subcategories: ‘Academic sponsor,’ ‘Industry sponsor,’ ‘Government sponsor,’ ‘Non-profit sponsor,’ and ‘Miscellaneous sponsor,’ with each subcategory coded as 1 for presence and 0 for absence. Trials may have more than one sponsor type.

Each treatment was assigned to exactly one of ten ranked categories: ‘Immunotherapy,’ ‘Antibody-drug conjugate (ADC),’ ‘Antibody,’ ‘Radiotherapy,’ ‘Hormone,’ ‘Targeted,’ ‘Chemotherapy,’ ‘Immune-Other,’ ‘Other,’ and ‘Supportive.’ A similar procedure was used to assign treatments in^45^. Details are in Supplementary Methods subsection SM4. In contrast to the sponsor types, a trial was assigned to the highest-ranked treatment type, The reason is that the highest-ranked treatment is usually the treatment being tested while other (lower-ranked) treatment types are often standard of care or supportive.

Start year of a trial was analyzed as a binary variable, partitioned at the median 2010, designated as early (started in or before 2010) and late (started after 2010). See Supplementary Figure S1. Details on analysis of primary variables can be found in Supplementary Methods subsection SM4.

### Threshold analysis of number of locations

Using Chi-squared tests, we compared trials with *z* or fewer ZIP codes to trials with more than *z* ZIP codes by their success rates, for *z* = 1, 5, 10, 20, 50, 100, 200, 500, and 700. Similarly, we compared trials with *s* or fewer states to trials with more than *s* states by their success rates for *s* = 1, 5, 10, 20 30, 40. We also performed polynomial fitting with polynomials of degrees 1,2,3,…,7, to model the relationship between the trial outcome and both “number of Distinct ZIP codes” and “number of Distinct states” variables in a fine-grained manner.

### Predictive modeling and statistical methods

All statistical analyses were performed using Python (version 3.12) libraries, including SciPy (scipy.stats), statsmodels (statsmodels.stats), and scikit-posthocs (scikit_posthocs). To assess associations and group differences, we used Chi-squared tests using scipy.stats.chi2_contingency for comparisons, Spearman correlation (using scipy.stats.spearmanr) for linear relationships between geographic features and success rate of trials, and the Kolmogorov–Smirnov test (scipy.stats.ks_2samp) to compare the distributions. For multiple group comparisons, Dunn’s test using scikit_posthocs.posthoc_dunn function was applied following Kruskal–Wallis tests (scipy.stats.kruskal). To control the false discovery rate (FDR) due to multiple comparisons, p-values from Dunn’s test were adjusted using the Benjamini-Hochberg approach via statsmodels.stats.multitest. Mann–Whitney U tests for pairwise comparisons were done using scipy.stats.mannwhitneyu. Odds ratios were calculated using statsmodels.stats.contingency_tables.Table 2 × 2 function. We reported odds ratios with 95% confidence intervals to describe effect sizes, noting whether the interval excludes 1.00; however, statistical significance was determined independently using the Kruskal–Wallis test followed by the post-hoc Dunn’s test. For all statistical tests that allowed for a choice between one-sided and two-sided options, the two-sided test was used. Statistical significance was assessed at a threshold of *p* < 0.05 unless otherwise specified.

For regression models, the continuous outcome variable in the range [0,1] was the success rate of trials in a ZIP code for ZIP codes with > 5 trials. We started with univariate regression. and added one variable at a time for two-variable and three-variable regression.

We generated models to predict trial success and used those models to assess the informational yield of geographic features in machine learning methods from the python library scikit-learn^52^. Among the machine learning methods we tested, random forest performed best. We defined trial success as a binary outcome (success = 1 or failure = 0). We implemented a Random Forest (RF) classifier using the final dataset of 15,658 trials partitioned five times into different training/testing subsets and we reported the average AUC of the five runs. We calculated feature importance for each version of our model and incrementally added features in descending order of importance where models used multiple features. Hyperparameter tuning was carried out using the GridSearchCV method in scikit-learn.

To evaluate predictive performance of these models, we used metrics such as Mean Absolute Error (MAE), Root Mean Square Error (RMSE), R^2^ (Coefficient of Determination), Spearman correlation and Pearson correlation between actual and predicted values of success rate. For machine learning models, we compared nested models that differ by the inclusion/exclusion of one variable or a set of variables by DeLong’s tests^38^.

In addition, we also conducted multivariable logistic regression analyses. These models evaluated geographic variables, non-geographic variables, and their combination to assess adjusted associations and potential confounding.

## Supplementary Information

Below is the link to the electronic supplementary material.

Supplementary Material 1

Supplementary Material 2

Supplementary Material 3

## Data Availability

Summary data for the central analyses in the manuscript are provided in Supplementary Tables S1, S2, and S7. The raw data for all trials analyzed in this study are available upon request to the corresponding author.
