# Supplementary material for: Success rates of American clinical oncology trials by geographic factors
Source: Sci Rep. 2026 Feb 11;16:8353. doi: 10.1038/s41598-026-39609-x (PMC12966375; doi:10.1038/s41598-026-39609-x)
Supplement: Supplementary file 1 — Supplementary Material 1 [file 41598_2026_39609_MOESM1_ESM.pdf]

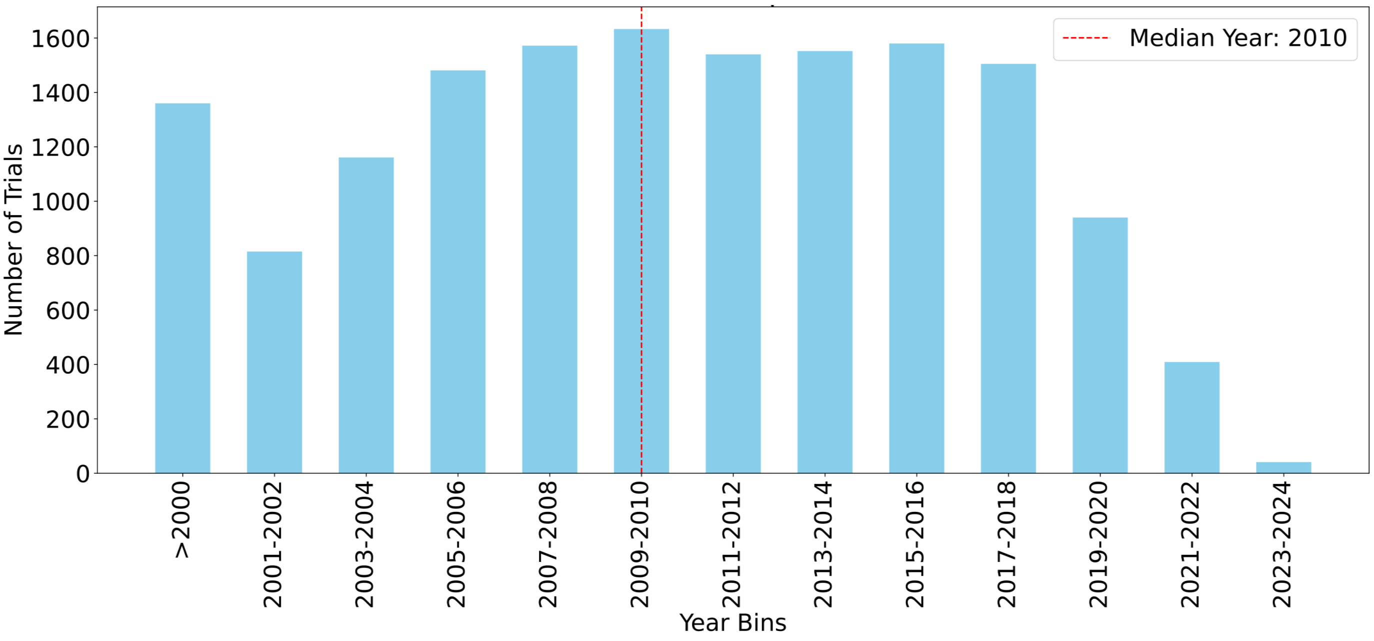

Supplementary Figure S1: **Distribution of clinical trials by bins of two consecutive start years (x-axis), showcasing the number of trials conducted over different time periods.** The dashed red line marks the median trial start year of 2010.

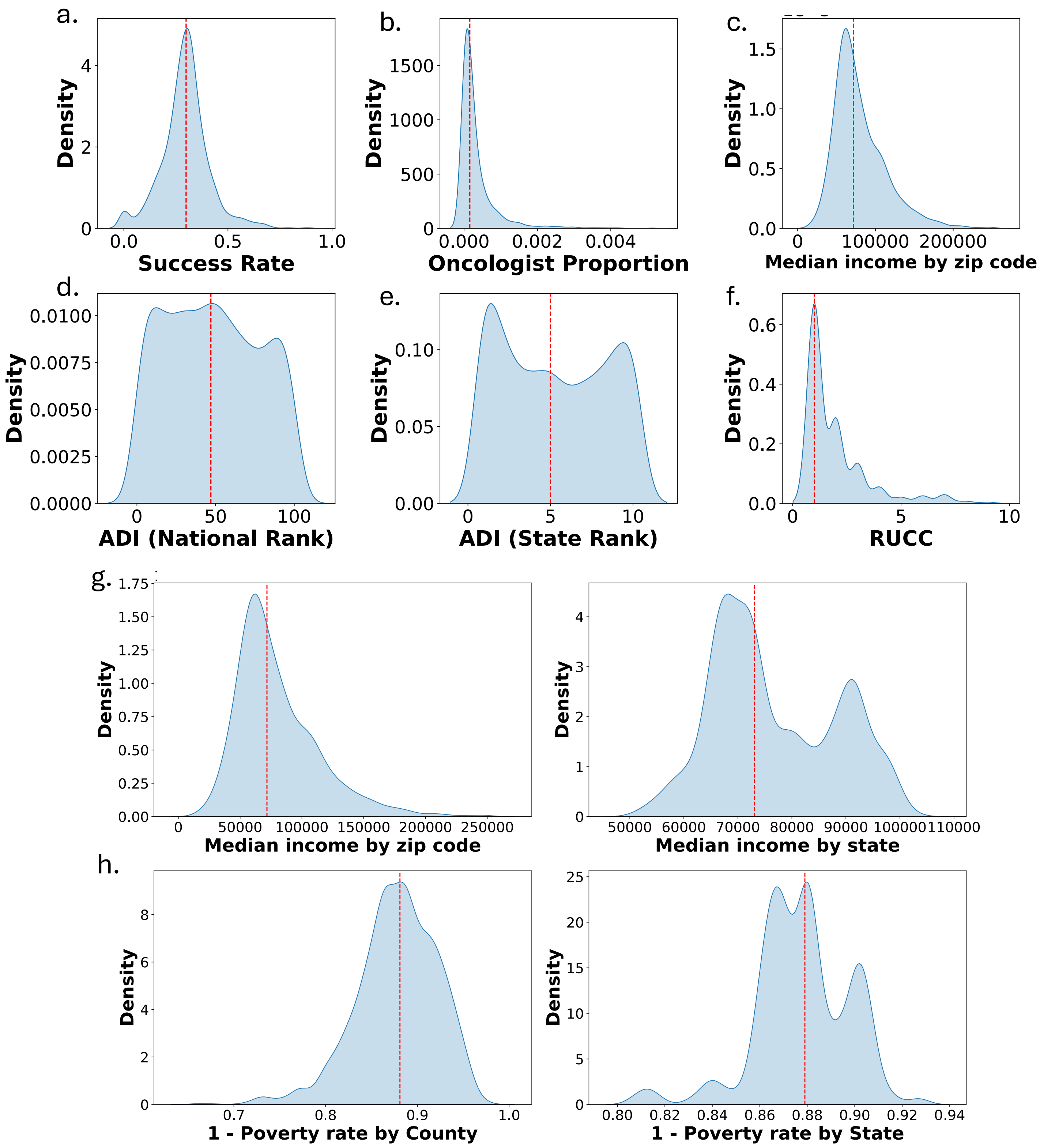

Supplementary Figure S2: **Density plots illustrating the distributions of variables influencing clinical trial outcomes restricted to ZIP codes with six or more trials.** (a) Success rate by ZIP code. (b) Oncologist proportion by ZIP code, depicting substantial variance with a peak at zero. (c) Median income by ZIP code. (d) ADI (National Rank) demonstrating a bimodal distribution. (e) ADI (State Rank) with a similar bimodal pattern. (f) RUCC (Rural-Urban Continuum Codes), showing the density of rural and urban classifications, underscoring geographical diversity in trial locations. (g) Density plots of median income at ZIP code and state levels, illustrating more variation at the ZIP code level compared to the state level. (h) Density plots of 1-poverty rate by county and state, revealing greater variation in 1-poverty levels at the county level.

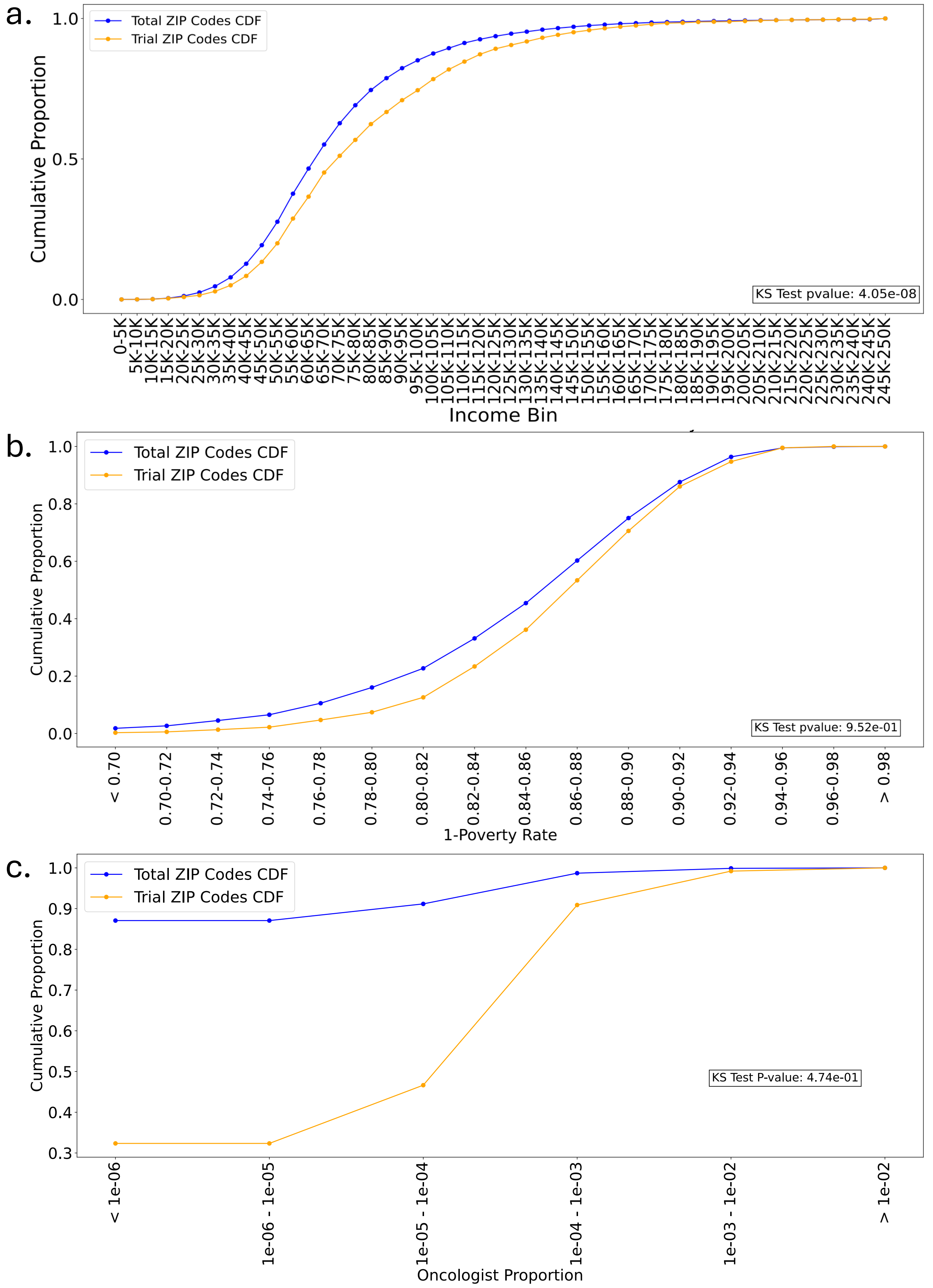

Supplementary Figure S3: **Cumulative distribution functions (CDFs) comparing the overall pool of ZIP codes (or counties) in the U.S. (blue) versus those involved in clinical trials (orange) for** (a) median income of ZIP codes, (b) $1 -$ poverty rate at the county level, and (c) oncologist proportion across ZIP code.

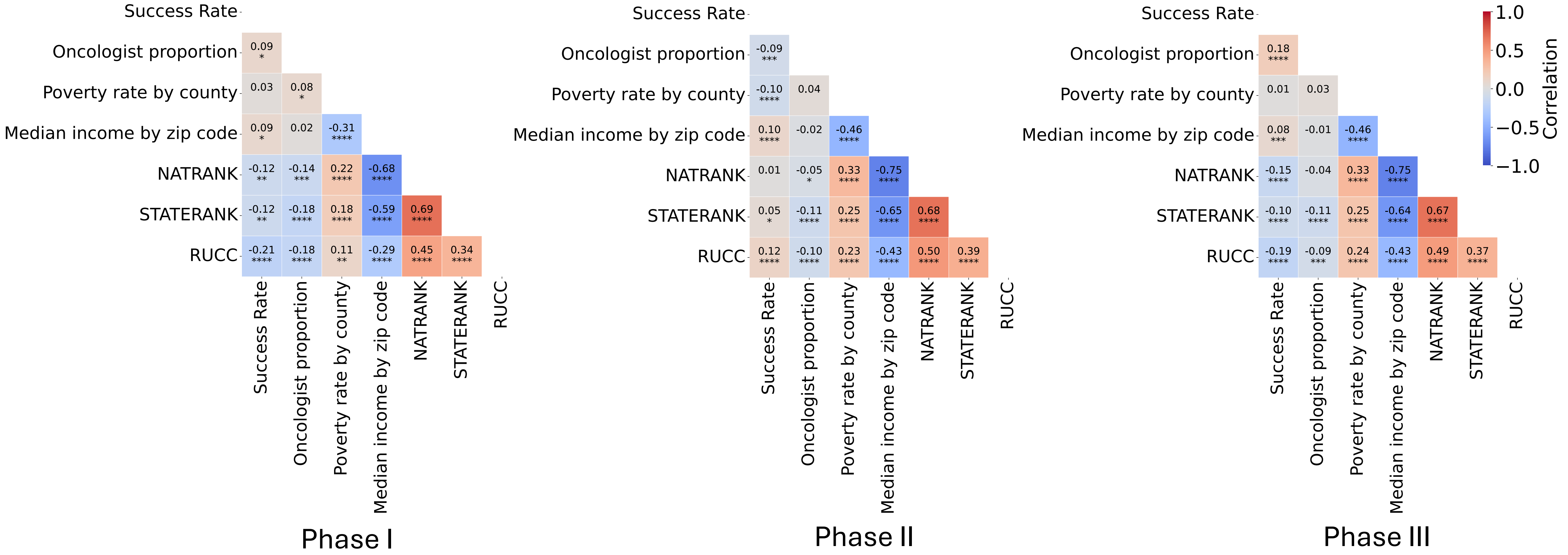

Supplementary Figure S4: **Specialization of the correlation analysis in main Figure 3a to trials of phases I, II, and III from left to right.** Spearman correlation matrices illustrating the relationships between success rate and key variables, including oncologist proportion by ZIP code, poverty rate by county, median income by ZIP code, ADI ranks (national and state) by ZIP code, and RUCC by county.

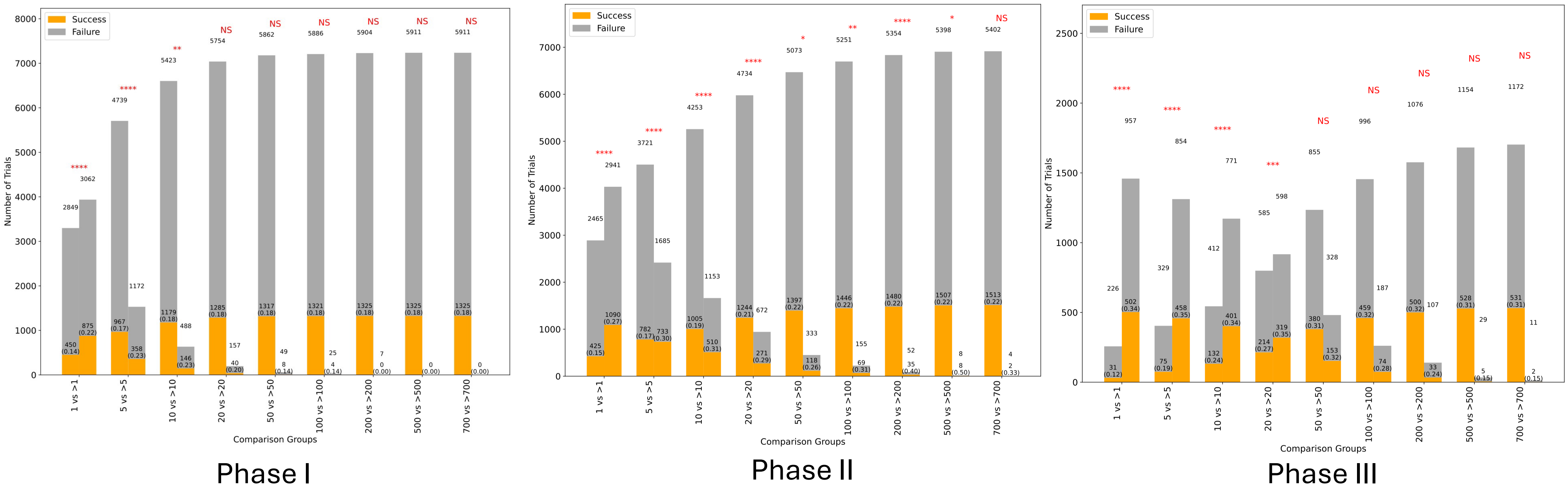

Supplementary Figure S5: **Specialization of the analysis in main Figure 3b to trials of phases I, II, III.** Comparison of success rates across trials involving fewer vs. more ZIP codes at various thresholds (x-axes) highlighting a trend of higher success rates with increased number of ZIP codes.

a.

b.

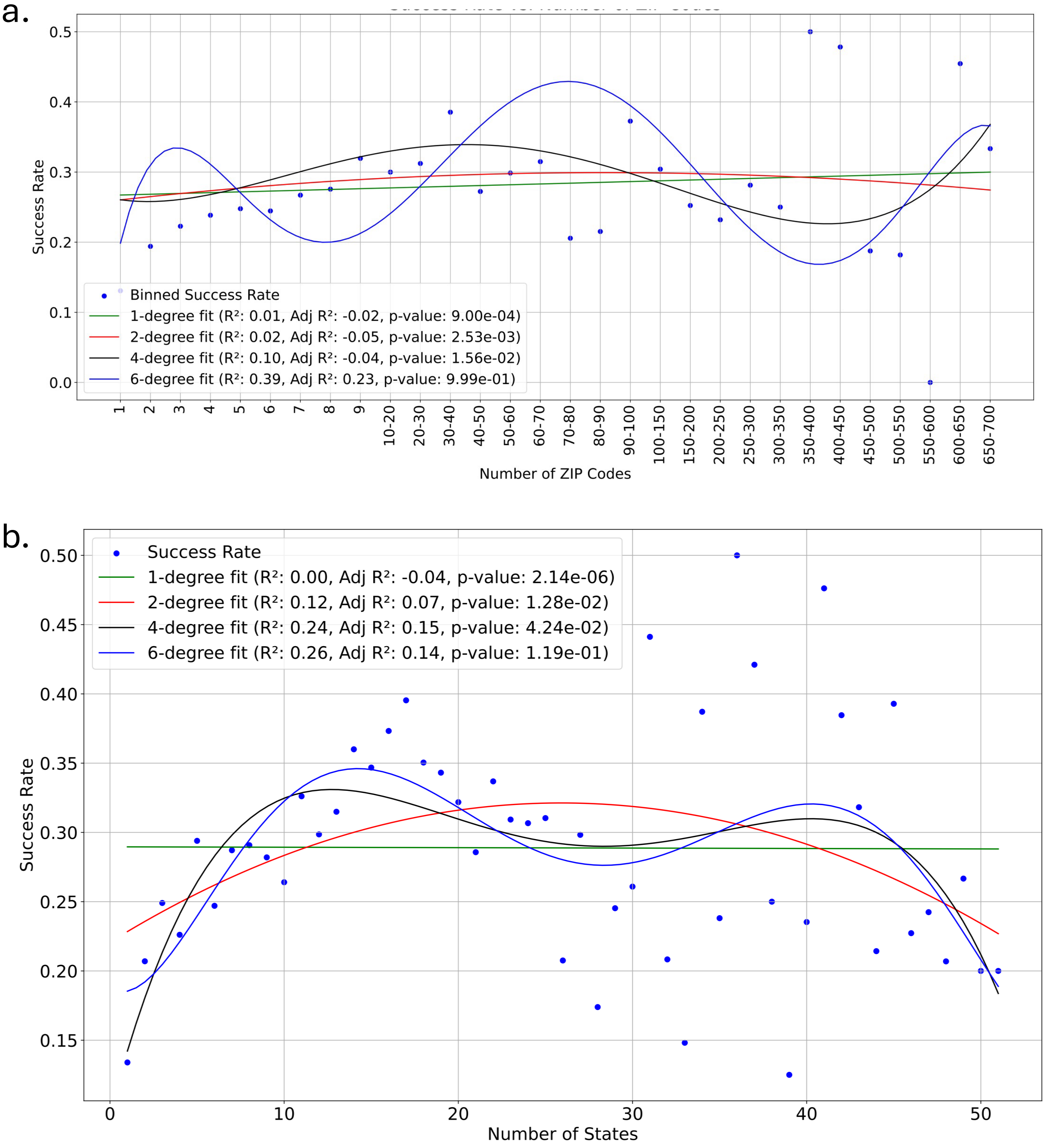

Supplementary Figure S6: **Impact of the number of sites on clinical trial outcomes.** (a) Polynomial fits, including linear and non-linear models, for the number of ZIP codes show weak trends, with success rate. (b) Polynomial fits, including linear and non-linear models, for the number of states reveal weak relationship with success rate.

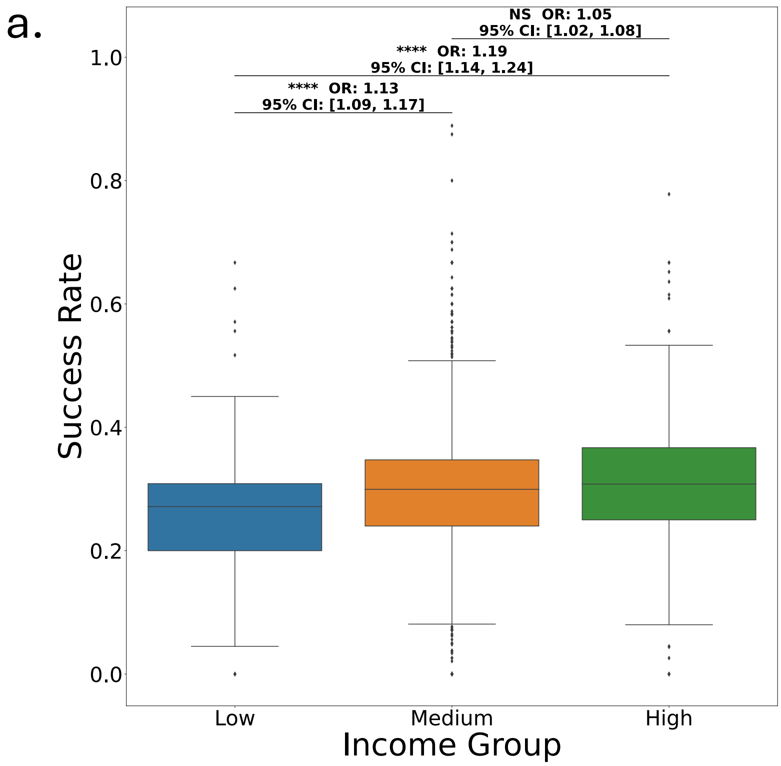

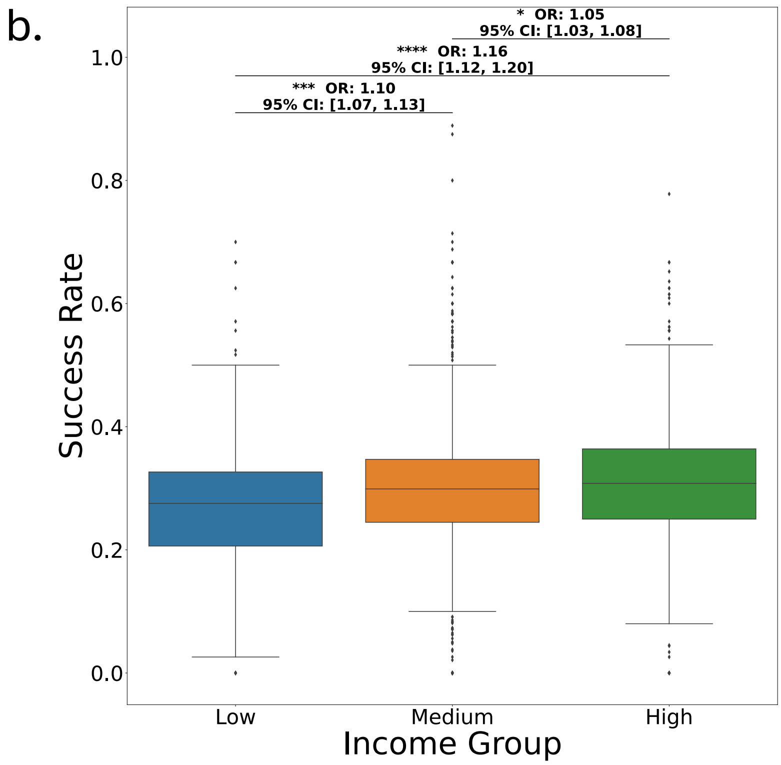

Supplementary Figure S7**: Boxplots showing the success rate across income groups categorized by different percentile thresholds.** (a) Income groups defined by the 10th and 90th percentiles: Low (<10th percentile), Medium (10th-90th percentile), and High (>90th percentile). (b) Income groups defined by 20th and 80th percentiles : Low (<20th percentile), Medium (20th-80th percentile), and High (>80th percentile). Odds ratios (OR) and 95% confidence intervals (CI) are displayed for pairwise comparisons between groups, highlighting differences in success rates across income levels. The p-values are from Kruskal-Wallis tests.

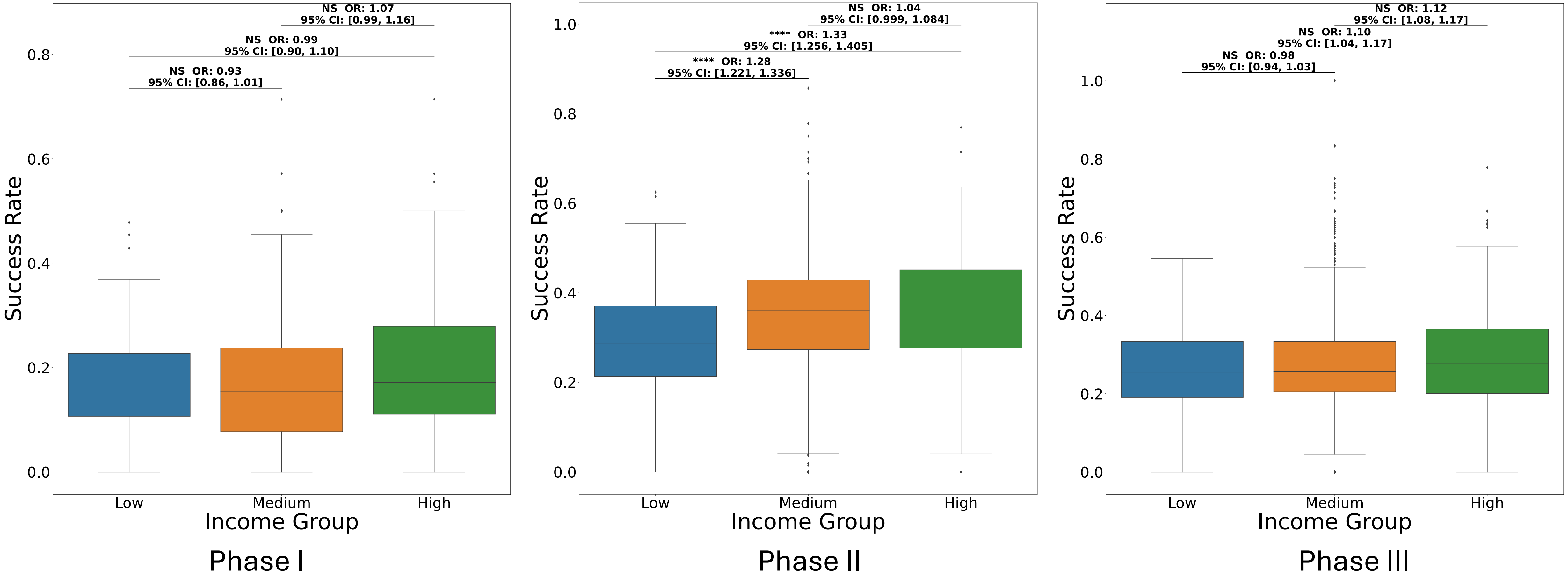

Supplementary Figure S8: **Specialization of the analysis in main Figure 3c to trials of phases I, II, III.** Box plots of success rates for ZIP codes categorized into low (<10th percentile), medium (10th–90th percentile), and high (>90th percentile) median income by ZIP code. Comparisons between groups via Kruskal-Wallis tests.

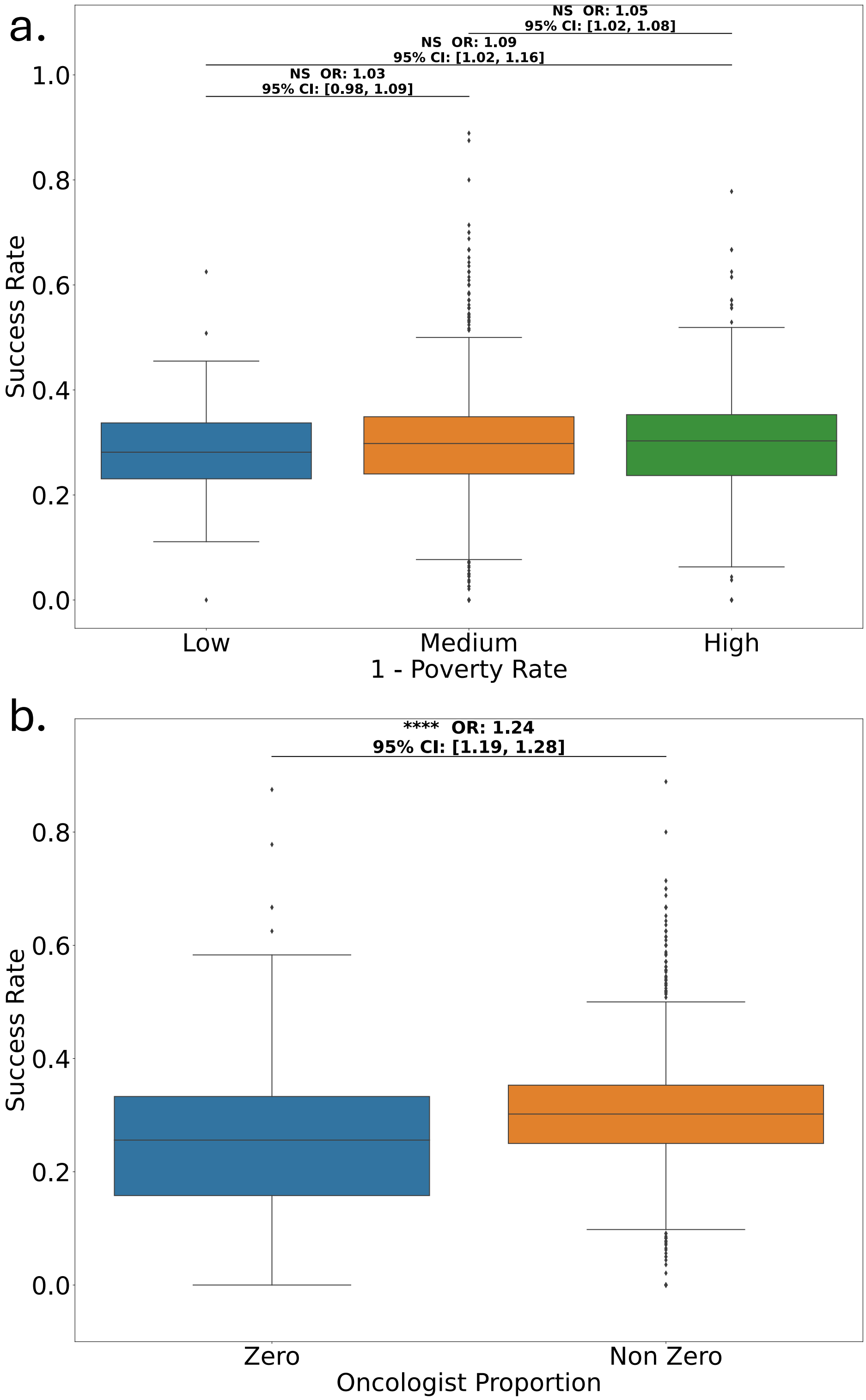

Supplementary Figure S9: **Comparison of success rates across different economic and healthcare-related categories.** (a) Success rates stratified by 1-poverty rate categories as Low (less than 10 percentile), Medium (between 10th and 90th percentile), and High (more than 90th percentile) show no statistically significant differences among the groups (NS). (b) Success rates for regions with Zero vs. non-zero oncologist proportion indicate a slightly higher success rate in regions with non-zero oncologist proportion, as reflected in the odds ratio (OR = 1.24, 95% CI: [1.19, 1.28]).

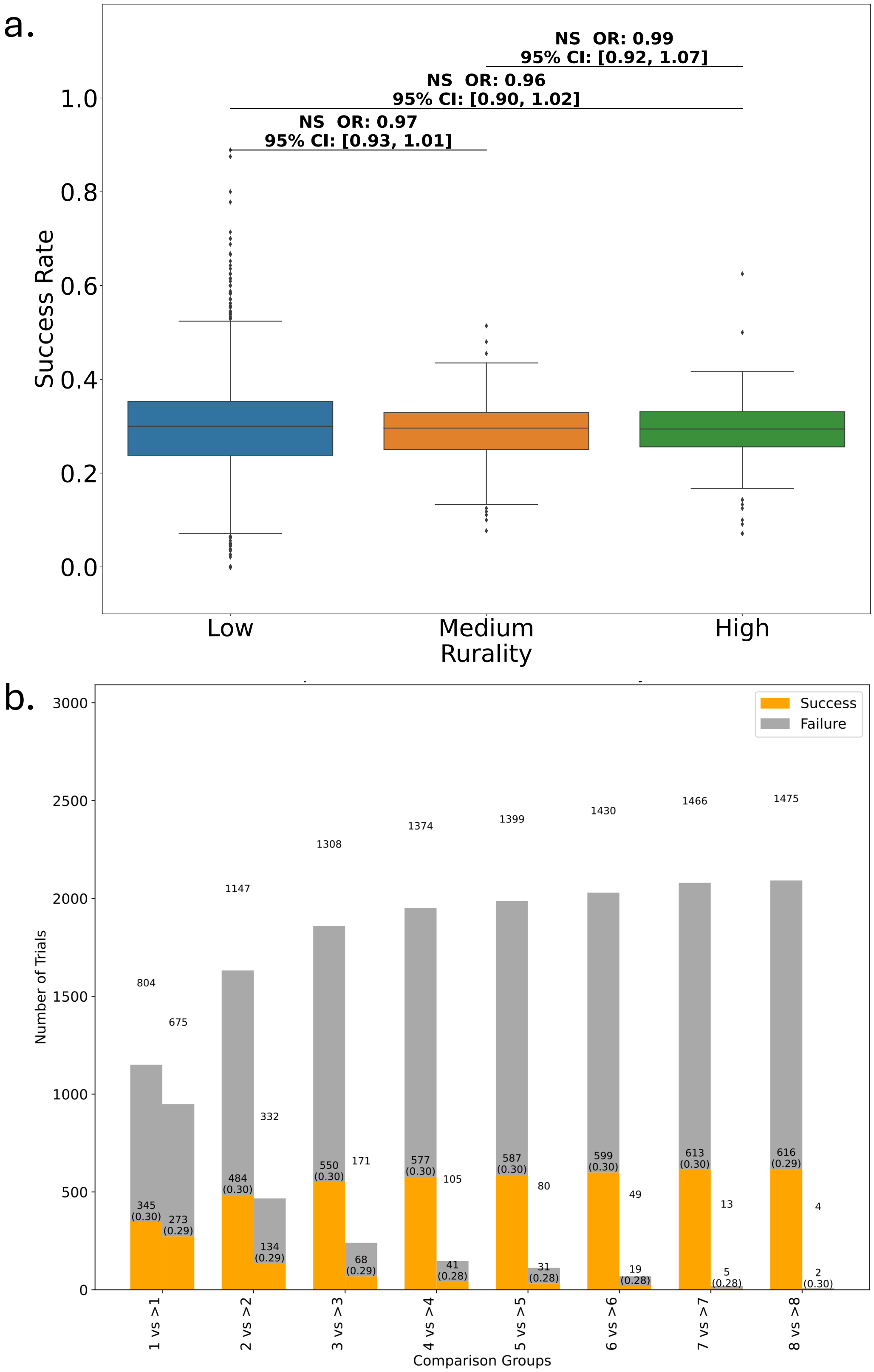

Supplementary Figure S10: **Analysis of success rates by rurality categories and RUCC.** (a) Box plots of clinical trial success rates by three RUCC-based rurality tiers—low (RUCC 1–3), medium (RUCC 4–6), and high (RUCC 7–9). (b) Bar charts comparing success and failure counts for trials grouped at progressively increasing RUCC thresholds (e.g., RUCC ≤ 1 vs. > 1, RUCC ≤ 2 vs. > 2, up to RUCC ≤ 8 vs. > 8).

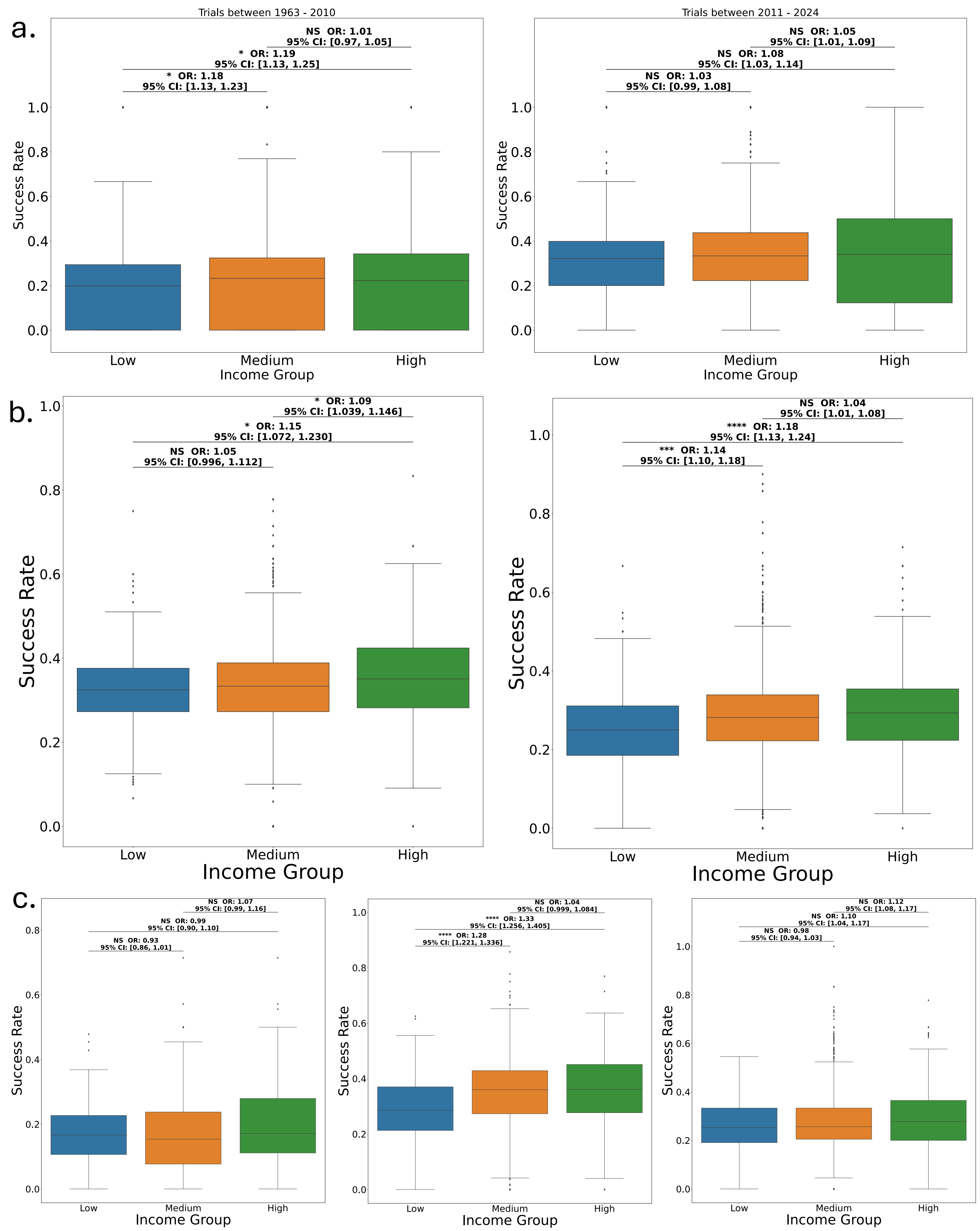

Supplementary Figure S11: **Boxplots of success rates across income groups categorized as Low (<10th percentile), Medium (10th–90th percentile), and High (>90th percentile), stratified by** (a) trial start year (1963–2010 vs. 2011–2024), (b) treatment type (Top 3 vs. Bottom 7), and (c) trial phase (I, II, III). Odds ratios (OR) with 95% confidence intervals (CI) are shown for pairwise comparisons, highlighting the variation in success rates across income groups within each stratification. The p-values are from Kruskal-Wallis tests

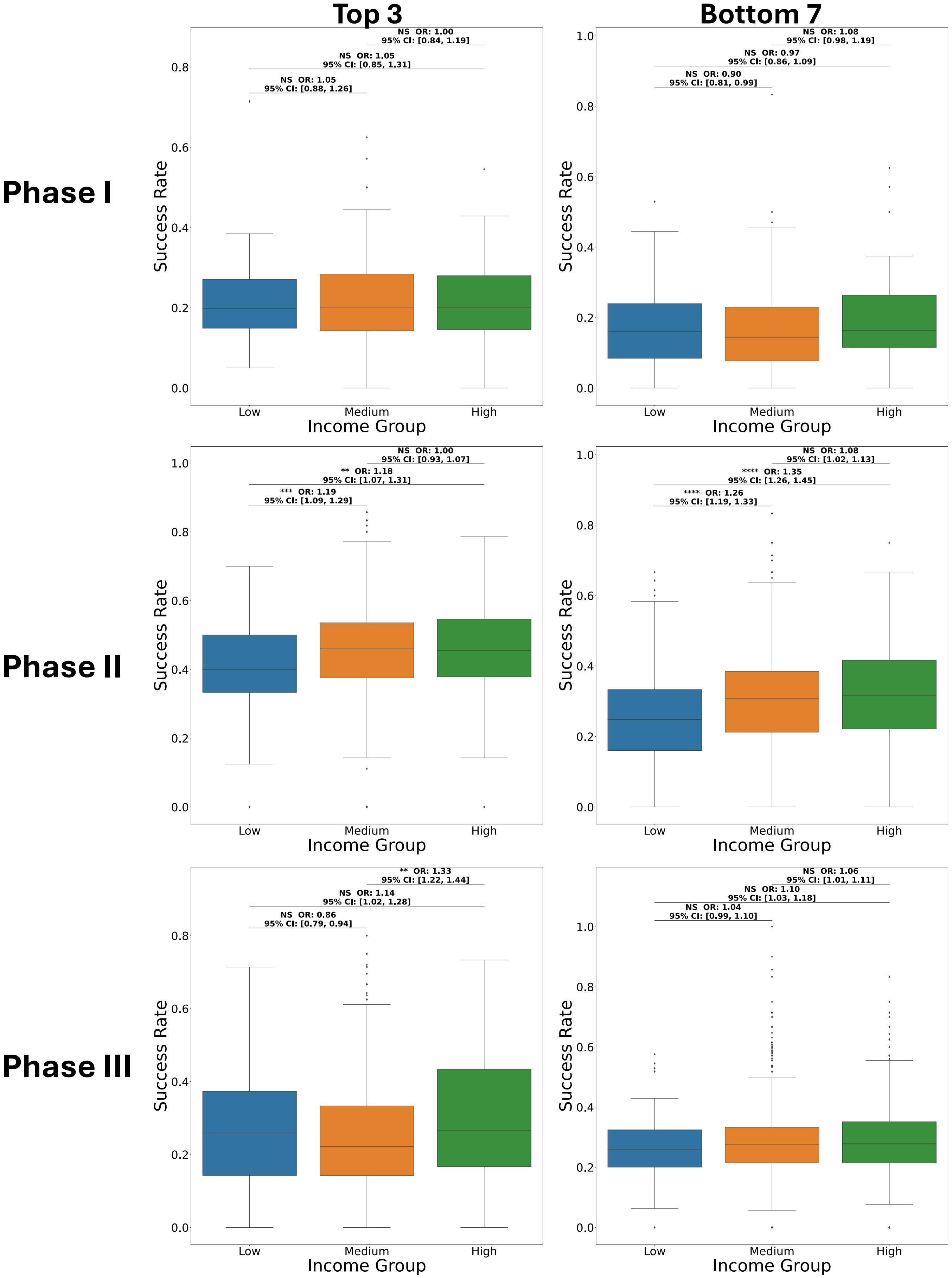

Supplementary Figure S12: **Boxplots of success rates across income groups categorized as Low (90th percentile), and combining the treatment types used in Supplementary Figure 8b and the phases used in Supplementary Figure 8c.** In this figure we compared the top three types to the bottom-ranked seven. The higher ranked treatments, such as immunotherapy and antibody-drug conjugates are more specialized. The full list of treatment types is in **Methods**

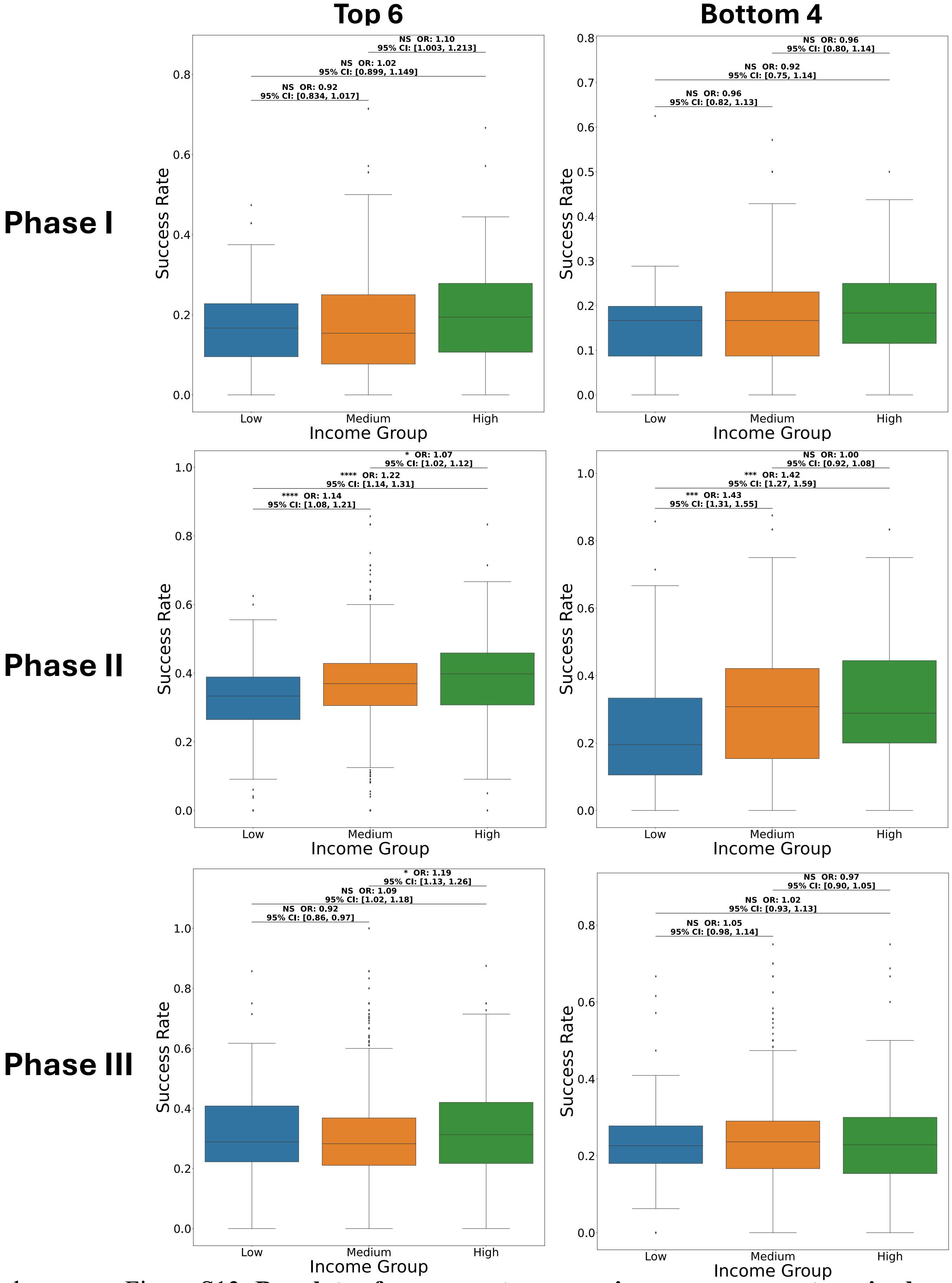

Supplementary Figure S13: **Boxplots of success rates across income groups categorized as Low (90th percentile), and combining the treatment types used in Supplementary Figure 8b and the phases used in Supplementary Figure 8c.** In this figure we compared the top-ranked six treatment types to the bottom-ranked four. The higher ranked treatments, such as immunotherapy and antibody-drug conjugates, are more specialized. The full list of treatment types is in **Methods**.

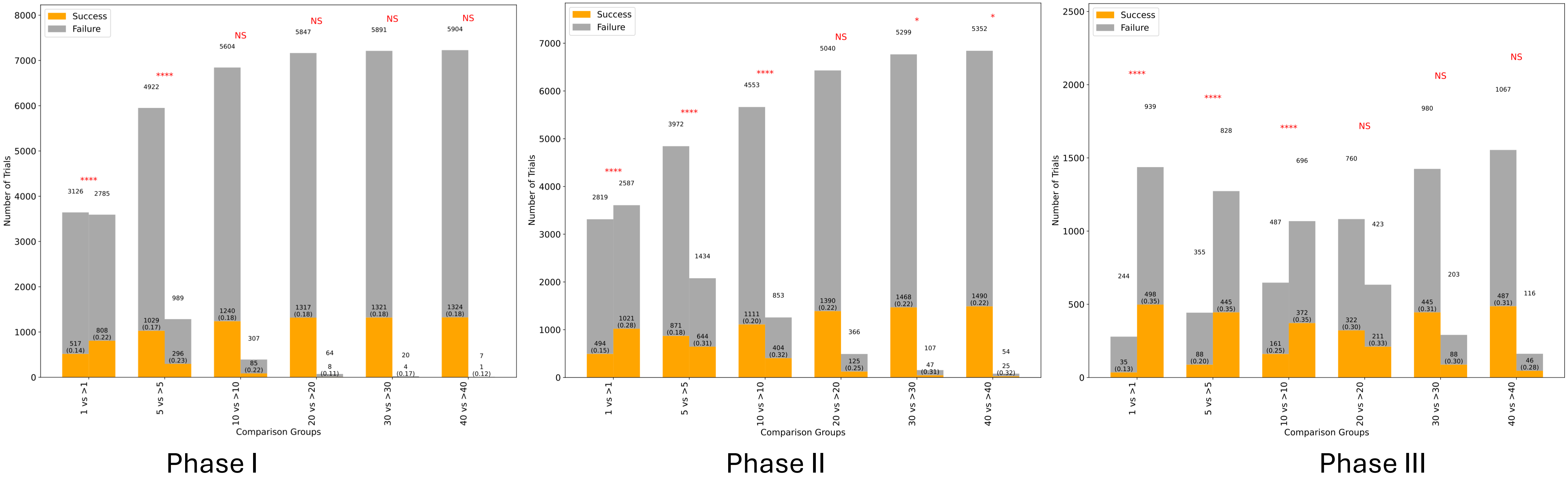

Supplementary Figure S14: **Specialization of the analysis in main Figure 3e to trials of phases I, II, III**. Comparison of success rates across trials involving fewer vs. more states codes at various thresholds (x-axes) highlighting a trend of higher success rates with increased number of states.
