# Supplementary material for: Success rates of American clinical oncology trials by geographic factors
Source: Sci Rep. 2026 Feb 11;16:8353. doi: 10.1038/s41598-026-39609-x (PMC12966375; doi:10.1038/s41598-026-39609-x)
Supplement: Supplementary file 2 — Supplementary Material 2 [file 41598_2026_39609_MOESM2_ESM.docx]

**Success rates of American clinical oncology trials by geographic factors**

**Sumeet Patiyal^1^, Alejandro A Schäffer^1^**

1. Cancer Data Science Laboratory, Center for Cancer Research, National Cancer Institute, Bethesda, MD 20892, USA.

**Supplementary Information**

**Supplementary Methods**

**S1. United States Geography Conventions and Some Unusual ZIP Code Mapping Cases**

At the highest level, the inhabited parts of the United States may be partitioned into 50 states, 1 District of Columbia, and 5 Territories (American Samoa, Guam, Northern Mariana Islands, Puerto Rico, U.S. Virgin Islands). Four of the 50 states (Kentucky, Massachusetts, Pennsylvania, Virginia) are officially called “commonwealths” rather than “states, but we ignored that distinction lumped the four commonwealths together with the 46 official ‘‘states”. For the reason explained in conjunction with Data source 1, in all subsequent text, “United States” refers only to the 50 states and the District of Columbia; in some places, we use “state” as shorthand for “state or District of Columbia”, but the District of Columbia is included in all analyses.

In addition to states, we collected information in four smaller geographic units: counties, cities, postal codes, and census tracts. Since 1963, each address in the U.S. has been assigned a postal code called a “ZIP code”, where “ZIP” stands for “Zone Improvement Plan”, a plan to improve the delivery of physical mail. We use the five-digit ZIP codes**.**

Usually, states are partitioned into counties to allow for more localized governance. Furthermore, within counties, there are many official cities, although some minimally inhabited areas are not in any city. In a different partition, the United States Census Bureau divides each state into census tracts, which are used for the census conducted every ten years (most recently in 2020). How to map between pairs of {state, county, city, ZIP code} for populated ZIP codes is explained carefully in publicly available data source 4 and the two associated references.

In most of the United States there is a hierarchy among ZIP codes, cities, and counties such that a ZIP code is in a unique city and a city is in one or more counties. However, there are a variety of exceptions to this nesting property. Three major US cities, namely Washington, D.C., Baltimore, and St. Louis, are not in any county; the commonwealth of Virginia also has several cities, such as Alexandria, that are not in any county. In cases where a city in multiple counties, we tried to assign that city to the county with the highest number of residents in the city; e.g., we assigned Houston, TX to Harris County. One exception is New York City, which is partitioned into five boroughs (Bronx, Brooklyn, Manhattan, Queens, Staten Island) each of which is a separate county. We treated the five New York City boroughs and their counties as separate entities when doing county-level analysis; this did not cause a problem since we did not do city-level analysis. One other type of exception concerns the term ‘county’. The state of Louisiana has ‘parishes’ instead of counties. The state of Alaska has ‘boroughs’ and ‘municipalities’ instead of counties, while in other states such as New Jersey and Pennsylvania, the term ‘borough’ is approximately a synonym for ‘city’.

**S2. Data Sources, Integration, and Quality Control**

In this study, we have obtained and merged the data from the six sources. (i) Trialtrove distinguishes U.S. Territories (e.g., Puerto Rico) from the 50 states and the District of Columbia; therefore, “United States” refers only to the 50 states and the District of Columbia. Trialtrove is available by paid license, which limits us to publishing summary data. We performed a Trialtrove data freeze on December 20, 2023. There were 95,861 oncology trials, of which 23,531 met the following two conditions: i) at least one trial site is in the United States; ii) the trial is described in the ‘Protocol/Trial ID’ with at least one alphanumeric code that starts with NCT and is followed by eight digits, such as NCT00000703, which is an identifier for ClinicalTrials.gov (source 2). (ii) In the data from ClinicalTrials.gov obtained via downloads of individual trial json-formatted files and an in-house parser, we obtained and checked the ZIP codes for locations of trials in the United States. Hundreds of manual corrections were needed, for example for ZIP codes such as 02215 that were missing the leading ‘0’ and for ZIP codes that had two digits transposed. (iii) From the United States census data, we have obtained three files, such as, (a) B01003 containing population by census tract or by ZIP code; a few ZIP code level entries are blank due to low population, (b) B17001 containing the poverty rate by census tract or by county; poverty rate by ZIP code is not available; the poverty rate by county version of the Table is effectively missing all counties in Connecticut because when the data were collected, Connecticut was not partitioned into counties, and (c) B19013 containing median income by census tract or by ZIP code. (iv) Information regarding oncologist were obtained from Centers for Medicare and Medicaid Services; using the IQR method cited in main **Methods**, we eliminated 36 ZIP codes with the highest oncologist proportions, those > 0.005783, because they represent areas with many medical offices and few residents. (v) ADI was obtained from University of Wisconsin. The data currently publicly available are the relative ranks in a state (between 1 and 10) or in the USA as a whole (between 1 and 100) for each ZIP code; these are denoted STATERANK and NATRANK, respectively. (vi) RUCC were obtained from U. S. Department of Agriculture.

We have used the two files to integrate the geographic entities obtained from U.S Department of Housing and Urban Development (HUD), such as, ZIP_TRACT_032024.xlsx to map between ZIP codes and census tracts, and ZIP_COUNTY_032024.xlsx and COUNTY_ZIP_032024.xlsx for mapping between counties and ZIP codes.

We also considered using data from https://cancercontrol.cancer.gov/hdhe/research-emphasis/underserved-areas, which provides binary data on whether counties qualify as a health professional shortage area (HPSA), qualify as non-metro, qualify as high poverty, and qualify as persistent poverty. We also considered using data from https://cancercontrol.cancer.gov/hdhe/research-emphasis/underserved-areas, which provides a list of ZIP codes that qualify as “far and remote (FAR)”. Ultimately, we decided not to use the HPSA or FAR data because the binary nature of these data makes them too coarse compared to the data sources we ultimately used.

**S3. Selection of Trials**

As illustrated in Figure 1b, we removed trials with no outcome, trials conducted entirely outside the 50 states and the District of Columbia, trials that are not in ClinicalTrials.gov (i.e., lack an alphanumeric code that starts with NCT), trials that lack usable ZIP code information in ClinicalTrials.gov, and trials that have an indeterminate outcome to get to 18,876 oncology clinical trials. For analyses that used phase, sponsor type, or treatment type, we additionally omitted 3,003 trials for which any of these three pieces of information was either blank or not usable (i.e., a phase of “Other”). For analyses using the start year of each trial, we omitted an additional 215 trials lacking the start year. The values of outcome, phase, sponsor type, and treatments are at least as complete in Trialtrove as in ClinicalTrials.gov, so we used the information from Trialtrove. These filtering steps got us to a final data set containing 15,658 trials.

**S4. More Details on Primary Variables for Data Analysis**

This subsection expands on main **Methods** subsection entitled “Primary Variables for Data Analysis”. We removed 36 ZIP codes that had the highest oncologist proportion as outliers. The preferred income measure does not have outliers at the upper end because values are capped at $250,000 by the U.S. Census Bureau. Although, we did not use the HPSA measures of poverty as primary variables (see above), we did use HPSA to cross-check the ACS data. We found that all but one county identified as a poverty area in HPSA has a county poverty estimate > 0.14, suggesting that 1 - poverty rate by county containing the ZIP code is a reasonable primary variable for a ZIP code.

In case of trial phase types, trials have one of eight possible values for phase: I, I/II, II, II/III, III, III/IV, IV, Other, with I, II, III being far more common than the other five values. To avoid using the uncommon values, we pooled I, I/II in phase I, pooled II, II/III in phase II, and pooled III, III/IV, IV in phase III. We dropped trials of phase “Other” because “Other” is not an ordinal value.

Each treatment was assigned to exactly one of the ten categories ‘Immunotherapy,’ ‘Antibody-drug conjugate (ADC),’ ‘Antibody,’ ‘Radiotherapy,’ ‘Hormone,’ ‘Targeted,’ ‘Chemotherapy,’ ‘Immune-Other,’ ‘Other,’ and ‘Supportive.’ based on information in Trialtrove and Google searches of various resources such as the National Cancer Institute (NCI) Dictionary of Terms, and the expertise of P.S.R. ‘Immunotherapy’ refers to immune checkpoint blockade treatments such as ipilimumab or pembrolizumab. ‘Antibody’ is used for treatments, such as cetuximab and rituximab, that use antibodies but are not ADCs. ‘Immune-other’ is used for treatments, such as cancer vaccines and bone marrow transplants, that directly manipulate the immune system, but are not immunotherapy, ADCs, or antibodies. In this context, “Radiotherapy” refers mostly to new, sophisticated radio-labeled drugs not radiation, which has been a mainstay of cancer treatment for decades, which explains why Radiotherapy is ranked highly (fourth). In general, the higher rankings informally represent more specialized treatments that are closer to our perception of the forefront of cancer treatments, except that the eighth-ranked ‘Immune-Other’ is a big mixture of treatment types, some quite new and some existing for decades and the ninth-ranked ‘Other’ is also a mixture of treatments, such as natural products, that are intended as primary treatments but do not fit in the other nine categories.

The most studied treatment type was Targeted therapy (32.54%), followed by Chemotherapy (19.84%) and Antibody (11.74%), reflecting current trends in therapeutic development. One-hot encoding was applied in the prioritized order, with each trial assigned a 1 for the highest-priority treatment and 0 for all others.

We used the selected variables such as “oncologist proportion”, “median income by ZIP code” and “poverty rate by county” containing the ZIP code individually and in combination to predict the success rate by applying various linear and non-linear regressors from the python library scikit-learn. To evaluate the performance, we used different metrices such as Mean Absolute Error (MAE), Root Mean Square Error (RMSE), R2 (Coefficient of Determination), Spearman rank correlation and Pearson correlation between actual and predicted values of the success rate.

We conducted an analysis to investigate the relationship between economic factors such as “median income by ZIP code,” “poverty rate by county”, “oncologist proportion” and clinical trial success rate, focusing on the distinction between “rich” and “non-rich” areas based on “median income by ZIP code” distribution. For this analysis, we considered only ZIP codes with more than five clinical trials, resulting in 2418 ZIP codes. To classify the ZIP codes into three parts of “rich”, “middle-income” and “poor” areas, we initially partitioned “median income by ZIP code” into equal thirds. To test the robustness of our results to the partition by income, we also tried 20-60-20 and 10-80-10 partitions. Pearson and Spearman correlation analyses were performed to assess the association between “median income by ZIP code,” “poverty rate by county,” “oncologist proportion”, ADI, RUCC one at a time with clinical trial “success rate”. We also did the income-based analysis separately for phases I, II, III.

To assess the relationship between success rate and the number of sites involved in clinical trials, we analyzed both the number of distinct ZIP codes and distinct states where trials were conducted. Our hypothesis suggests that a moderate number of sites (ZIP codes or states) is most effective, balancing the need for sufficient patient enrollment with manageable site coordination. We utilized two variables. First, we did a coarse dichotomous test comparing trials with at most s sites and trials with more than s sites, e.g., if s = 10, we compare either proportion of successful trials in 10 or fewer ZIP codes or states to proportion of successful trials in >10 ZIP codes or states. We also did this analysis separately for phases I, II, III. We also performed polynomial fitting with polynomials of degrees 1,2,3,…,7, to model the relationship between the trial outcome and both “Distinct ZIP codes” and “Distinct states” variables in a fine-grained manner. This approach allowed us to investigate the non-linear trends.

We developed multiple models to predict the success of clinical trials using both geographic and non-geographic features. We defined trial success as a binary outcome (success = 1 or failure = 0). We implemented a Random Forest (RF) classifier using the final dataset of 15,658 trials (see subsections SM2 and SM3 above and **Figure 1b**) partitioned 5 times into different training/testing subsets. We calculated feature importance for each version of our model, and incrementally added features in descending order of importance where models used multiple features. Hyperparameter tuning was carried out using the GridSearchCV method in scikit-learn.

**Legends of Supplementary Figures**

Supplementary Figure S1: **Distribution of clinical trials by bins of two consecutive start years (x-axis), showcasing the number of trials conducted over different time periods.** The dashed red line marks the median trial start year of 2010.

Supplementary Figure S2: **Density plots illustrating the distributions of variables influencing clinical trial outcomes restricted to ZIP codes with six or more trials.** (a) Success rate by ZIP code. (b) Oncologist proportion by ZIP code, depicting substantial variance with a peak at zero. (c) Median income by ZIP code. (d) ADI (National Rank) demonstrating a bimodal distribution. (e) ADI (State Rank) with a similar bimodal pattern. (f) RUCC (Rural-Urban Continuum Codes), showing the density of rural and urban classifications, underscoring geographical diversity in trial locations. (g) Density plots of median income at ZIP code and state levels, illustrating more variation at the ZIP code level compared to the state level. (h) Density plots of 1-poverty rate by county and state, revealing greater variation in 1-poverty levels at the county level.

Supplementary Figure S3: **Cumulative distribution functions (CDFs) comparing the overall pool of ZIP codes (or counties) in the U.S. (blue) versus those involved in clinical trials (orange) for** (a) median income of ZIP codes, (b) 1 − poverty rate at the county level, and (c) oncologist proportion across ZIP codes.

Supplementary Figure S4. **Specialization of the correlation analysis in main Figure 3a to trials of phases I, II, and III from left to right.** Spearman correlation matrices illustrating the relationships between success rate and key variables, including oncologist proportion by ZIP code, 1-poverty rate by county, median income by ZIP code, ADI ranks (national and state) by ZIP code, and RUCC by county.

Supplementary Figure S5: **Specialization of the analysis in main Figure 3b to trials of phases I, II, III.** Comparison of success rates across trials involving fewer vs. more ZIP codes at various thresholds (x-axes) highlighting a trend of higher success rates with increased number of ZIP codes.

Supplementary Figure S6: **Impact of the number of sites on clinical trial outcomes.** (a) Polynomial fits, including linear and non-linear models, for the number of ZIP codes show weak trends, with success rate. (b) Polynomial fits, including linear and non-linear models, for the number of states reveal weak relationship with success rate.

Supplementary Figure S7**: Boxplots showing the success rate across income groups categorized by different percentile thresholds.** (a) Income groups defined by the 10th and 90th percentiles: Low (<10th percentile), Medium (10th-90th percentile), and High (>90th percentile). (b) Income groups defined by 20th and 80th percentiles: Low (<20th percentile), Medium (20th-80th percentile), and High (>80th percentile). Odds ratios (OR) and 95% confidence intervals (CI) are displayed for pairwise comparisons between groups, highlighting differences in success rates across income levels. The p-values are from Kruskal-Wallis tests.

Supplementary Figure S8: **Specialization of the analysis in main Figure 3c to trials of phases I, II, III.** Box plots of success rates for ZIP codes categorized into low (<10th percentile), medium (10th–90th percentile), and high (>90th percentile) median income by ZIP code. Comparisons between groups via Kruskal-Wallis tests.

Supplementary Figure S9: **Comparison of success rates across different economic and healthcare-related categories.** (a) Success rates stratified by 1-poverty rate categories as Low (less than 10 percentile), Medium (between 10^th^ and 90^th^ percentile), and High (more than 90^th^ percentile) show no statistically significant differences among the groups (NS). (b) Success rates for regions with Zero vs. non-zero oncologist proportion indicate a slightly higher success rate in ZIP Codes with non-zero oncologist proportion, as reflected in the odds ratio (OR = 1.14, 95% CI: [0.02, 54.63]). Many trials have sites with zero oncologist proportion since oncologists can move from one ZIP Code to another to deliver care.

Supplementary Figure S10: **Analysis of success rates by rurality categories and RUCC.** (a) Box plots of clinical trial success rates by three RUCC‐based rurality tiers—low (RUCC 1–3), medium (RUCC 4–6), and high (RUCC 7–9). (b) Bar charts comparing success and failure counts for trials grouped at progressively increasing RUCC thresholds (e.g., RUCC ≤ 1 vs. > 1, RUCC ≤ 2 vs. > 2, up to RUCC ≤ 8 vs. > 8).

Supplementary Figure S11: **Boxplots of success rates across income groups categorized as Low (<10th percentile), Medium (10th–90th percentile), and High (>90th percentile), stratified by** (a) trial start year (1963–2010 vs. 2011–2024), (b) treatment type (Top 3 vs. Bottom 7), and (c) trial phase (I, II, III). Odds ratios (OR) with 95% confidence intervals (CI) are shown for pairwise comparisons, highlighting the variation in success rates across income groups within each stratification. The p-values are from Kruskal-Wallis tests.

Supplementary Figure S12. **Boxplots of success rates across income groups categorized as Low (90th percentile), and combining the treatment types used in Supplementary Figure 11b and the phases used in Supplementary Figure 11c.** In this figure we compared the top three types to the bottom-ranked seven. The higher ranked treatments, such as immunotherapy and antibody-drug conjugates are more specialized. The full list of treatment types is in **Methods**.

Supplementary Figure S13. **Boxplots of success rates across income groups categorized as Low (90th percentile), and combining the treatment types used in Supplementary Figure 11b and the phases used in Supplementary Figure 11c.** In this figure we compared the top-ranked six treatment types to the bottom-ranked four. The higher ranked treatments, such as immunotherapy and antibody-drug conjugates, are more specialized. The full list of treatment types is in **Methods**.

Supplementary Figure S14. **Specialization of the analysis in main Figure 3e to trials of phases I, II, III**. Comparison of success rates across trials involving fewer vs. more states codes at various thresholds (x-axes) highlighting a trend of higher success rates with increased number of states.

**Supplementary Tables**

Supplementary Table S1. Distribution of successful and failed clinical trials across non-geographic features, categorized by trial phases (I, II, III), sponsor types (five categories), treatment types (ten categories) and start year.

Supplementary Table S2. Integrated data table summarizing information from multiple sources for ZIP codes with six or more clinical trials. The table is sorted in decreasing order by success rate.

Supplementary Table S3. Coefficients and Bonferroni-corrected p-values from linear regression models analyzing the relationship between success rate and key variables.

Supplementary Table S4. Performance of linear and non-linear regression models for predicting success rates using Median Income by ZIP Code and one additional variable. The table summarizes model types, evaluation metrics (MAE, RMSE, R²), and correlation values (Pearson and Spearman), with their respective p-values.

Supplementary Table S5. Potential trial site ZIP codes that have had no oncology clinical trials, have rurality threshold of RUCC at least 6, at least 1 practicing oncologist, and no NCI-designated cancer center within 50 miles.
